# Biomimetic cancer cell membrane-enriched vitamin E-stapled gemcitabine-loaded TPGS micelles for pancreatic cancer therapy

**DOI:** 10.1080/10717544.2025.2527759

**Published:** 2025-07-09

**Authors:** Miguel Pereira-Silva, Luis Diaz-Gomez, Bárbara Blanco-Fernandez, Ana Cláudia Paiva-Santos, Francisco Veiga, Angel Concheiro, Carmen Alvarez-Lorenzo

**Affiliations:** a Department of Pharmaceutical Technology, Faculty of Pharmacy, University of Coimbra, Coimbra, Portugal; b Faculty of Pharmacy, Group of Pharmaceutical Technology, REQUIMTE/LAQV, University of Coimbra, Coimbra, Portugal; c Departamento de Farmacología, Farmacia y Tecnología Farmacéutica, Facultad de Farmacia, I + D Farma, Instituto de Materiales (iMATUS) and Health Research Institute of Santiago de Compostela (IDIS), Universidade de Santiago de Compostela, Santiago de Compostela, Spain

**Keywords:** Polymeric micelle, vitamin E-based nanosystem, pancreatic cancer, chemotherapy, gemcitabine, biomimetic, cell membrane coating

## Abstract

Pancreatic cancer (PC) is currently a leading cause of death worldwide and its incidence is expected to increase in the following years. Chemotherapy with gemcitabine (GEM) is precluded by extensive enzymatic inactivation and clearance, and the nonspecific tissue distribution contributes to unwanted systemic toxicity and tumor resistance. In this work, GEM was encapsulated in d-ɑ-tocopheryl polyethylene glycol succinate (TPGS) micelles by ‘stapling’ GEM at 4-NH_2_ position with vitamin E succinate (VES) through a highly stable amide bond, achieving successful GEM hydrophobization by means of a prodrug system (VES–GEM). Recurring to solvent evaporation methodology, TPGS/VES–GEM (6/1 molar ratio) micelles were prepared, optimized regarding TPGS-to-VES–GEM ratio, and characterized regarding size, surface charge, polydispersity index, morphology, drug loading, and encapsulation efficiency (EE). Furthermore, purification methods were explored together with VES–GEM release profile and stability. Lastly, cell viability and cellular uptake of the formulation were analyzed in 2D and 3D BxPC3 cell line models. TPGS/VES–GEM micelles (6/1) showed ultra-small size (∼30 nm), and remarkable EE (>95%) together with ability to retain VES–GEM for long period of time (>7 days) with high stability. The micelles demonstrated exceptional cell cytotoxic activity for concentrations of 10 and 100 µM VES–GEM (∼0% cell viability) which may be explained by concerted action of GEM, VES, and TPGS. The nanocarrier was further enriched with PC cell membrane nanovesicles, displaying size ∼150 nm, ZP ∼ −30 mV and PDI ∼0.2 to improve biointerfacing properties and targeting properties. BxPC3 cell membrane-modified TPGS/VES–GEM micelles may be attractive biomimetic nanosystem for next-generation PC therapeutics.

## Graphical Abstract

**Figure:**
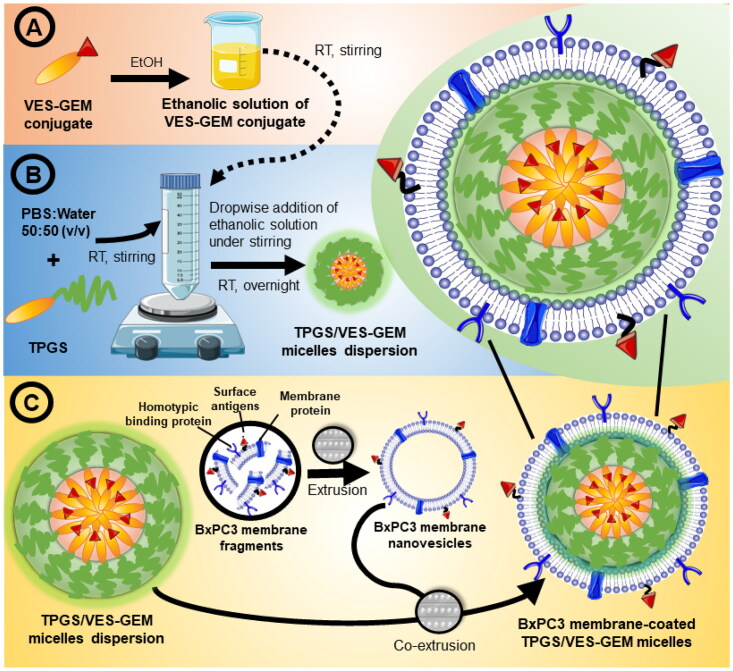

Schematic illustration of the preparation of TPGS/VES–GEM, and BxPC3 cell membrane-coated TPGS/VES–GEM micelles for GEM delivery to PC cells.

## 1. Introduction

With its aggressive nature, limited treatment choices and delayed detection, pancreatic cancer (PC) remains a clinical challenge in oncology (Buckley and O’Reilly 2024; Hu and O’Reilly 2024). Conventional treatment comprises chemotherapy, radiotherapy, and surgical removal of the tumor mass (Buckley and O’Reilly 2024; Hu and O’Reilly 2024). Although widely used as therapeutic modality in PC, chemotherapy – for instance, gemcitabine (GEM, 2′,2′-difluoro-2′-deoxycytidine) or drug cocktail FOLFIRINOX – displays limited efficacy and strong systemic toxicity. GEM itself is associated to several drawbacks, including extensive clearance, reduced blood circulation half-life, off-target toxicity, and lack of stability in serum (Han et al. 2022).

In this regard, nanocarriers may offer novel approaches for combating PC by enabling more precise and controlled drug administration and improve therapy effectiveness while minimizing side effects (Yan et al. 2023). Polymeric micelle-based drug delivery systems have been receiving increasing attention on account of their simplicity, ease of preparation, fine-tuned physicochemical properties, and small size (Zheng et al. 2024). Moreover, several building blocks used in preparation of micelles – including amphiphilic block copolymers like poloxamers (poloxamer 407) (Meng et al. 2017) and graft copolymers such as polyvinyl caprolactam–polyvinyl acetate–polyethylene glycol (Soluplus^®^) (Wang et al. 2020) – display biorelevant functions and are able to improve cancer therapy posing as functional excipients. PEGylated vitamin-based micelles, such as PEGylated vitamin D (Kutlehria et al. 2018) and PEGylated vitamin E (VE) (Kumbhar et al. 2022) are interesting synthetic vitamin derivatives with bioactive building blocks for micelles preparation and show anticancer properties and also function as vitamin prodrug reservoirs. d-ɑ-Tocopheryl polyethylene glycol succinate, also known as VE TPGS, is a bioactive water-soluble, low molecular weight (∼1513 g/mol) amphiphilic polymer composed of vitamin E succinate (VES) coupled to short PEG chain (PEG1000) (Rathod et al. 2021). It is included in FDA- and EMA-approved medicines, recognized as biocompatible and safe excipient, used as solubilizer, emulsifier, stabilizer, permeation enhancer, and antioxidant agent. TPGS can be administered through various routes, including parenteral administration, for a variety of therapeutic and cosmetic purposes (Tan et al. 2017; Yang et al. 2018; Mehata et al. 2023). Moreover, TPGS has critical micellar concentration of 0.132 mM (Rathod et al. 2021), or 0.02%w/w (Kumbhar et al. 2022), and is able to form stable micelles with small size (<20 nm) in aqueous medium (Rathod et al. 2021). It contains a hydrophilic head composed of PEG and a hydrophobic alkyl tail from VES. Besides serving as a structural unit for encapsulation of hydrophobic drugs in the core of micelles, it also possesses significant anticancer activities, namely multidrug resistance (MDR) reversal capability by inhibiting P-glycoprotein (P-gp) (Cheng et al. 2017; Liang et al. 2018). Other potential anticancer activities include the ability to amplify ROS production in cancer cells and induce apoptotic signaling and cancer cell death (Youk et al. 2005; Neophytou et al. 2014; Ruiz-Moreno et al. 2018; Chen et al. 2020; Tang et al. 2023). Additional antioxidant and anticancer activities result from cleavage of ester bond releasing redox-active VES (Grimaudo et al. 2018; Kumbhar et al. 2022). TPGS micelles have been shown to improve bioavailability of hydrophobic drugs by efficient drug encapsulation and protection, prolonged blood circulation half-life, enhanced stability, intrinsic bioactive features, and amenable controlled release profile (Zhang et al. 2012; Tan et al. 2017; Yang et al. 2018; Mehata et al. 2023). TPGS micelles were explored as delivery systems of GEM by coupling GEM to PEG chain assembling prodrug TPGS-GEM micelles (Khare et al. 2016), or employing TPGS to encapsulate lipid-GEM derivatives (Wang et al. 2014; Xu et al. 2015). TPGS can also be incorporated in other systems to improve stability and functionality (Chen et al. 2021; Yusuf et al. 2021). On the other hand, VES, also known as alpha-tocopheryl succinate (α-TOS), is a VE analogue that integrates TPGS structure and can also be employed separately in nanosystems as bioactive compound, bearing potent anticancer activity known to inhibit cancer growth through distinct mechanisms. Among its anticancer activities, it can reduce cell proliferation, act as apoptosis inductor in both extrinsic and mitochondria (intrinsic)-mediated pathways, and help preventing metastatic phenomena, target mitochondria with high specificity and contribute to MDR reversal (Patacsil et al. 2012; Angulo-Molina et al. 2014; Liang and Qiu 2021).

GEM loading in micelles faces several challenges; the main one being related to its hydrophilic character which precludes its successful encapsulation. Attempts to develop GEM-loaded micelles are sparse and still underexplored. The development of lipophilic GEM prodrugs has been explored before to reduce its systemic toxicity and enhance uptake by cancer cells, and VES has served as lipophilic block for establishing GEM prodrug derivative with enhanced bioactivities (Duhem et al. 2014; Guo et al. 2020). Recently, biomimetic nanosystems incorporating cancer cell membranes have shown to improve targeting features, immune evasion and blood circulation of nanoparticle cores (Pereira-Silva et al. 2020; Ma et al. 2023), including micelle-based ones (Zhao et al. 2023; Huang et al. 2024).

**Figure 1.**
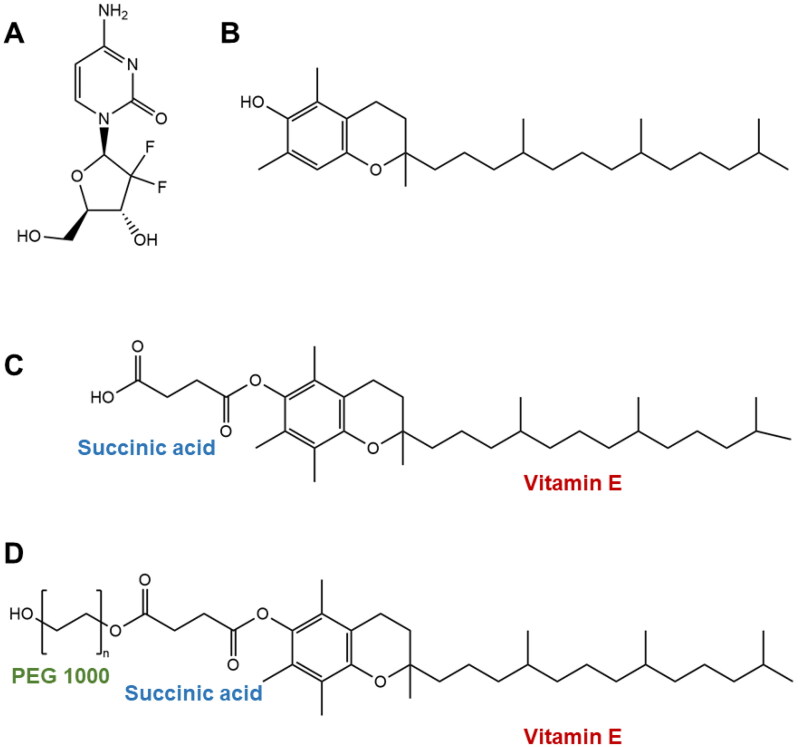
Chemical structures of gemcitabine (A), vitamin E (B), vitamin E succinate (C), and d-ɑ-tocopheryl polyethylene glycol succinate – TPGS (D).

In this work, a novel strategy was devised to facilitate GEM micellization in the hydrophobic core of VE-enriched micelles, thus assembling a micellar derivative of GEM. For that, GEM was modified with VES – *stapled* – (Figure 1(A–D)) in order to (1) confer hydrophobic properties and improve affinity with hydrophobic cavity of TPGS, (2) add anti-cancer biofunctional properties to GEM therapy, (3) improve stability of GEM by –NH_2_ group protection and through the formation of highly stable amide bond, (4) promote extra-long and controlled GEM release, and (5) build new avenues toward GEM micellization, particularly in TPGS micelles, without addition of co-surfactant and in the form of double-action prodrug with mechanistic versatility. The VE-stapled GEM-loaded TPGS micelles were prepared and characterized, and cellular internalization was assessed. Purification methods including centrifugation, filtration, and ultrafiltration were critically examined to assess encapsulation efficiency (EE) and stability parameters which provided more consubstantiated data often underestimated in previous reports. To leverage targeting and biomimetic features of the system, biomimetic PC cell membranes were extracted from BxPC3 cell line and employed to coat TPGS micelle cores, and the PC cell membrane-enriched VE-stapled GEM-loaded TPGS micelles were characterized regarding size, surface charge, polydispersity index (PDI), and morphology. Both BxPC3 cell membrane-enriched TPGS/VES–GEM and TPGS/VES–GEM micelles were further analyzed regarding anticancer activity by measuring cell viability using 2D BxPC3 cell line and 3D BxPC3-laden collagen hydrogels.

## 2. Materials and methods

### 2.1. Materials

Gemcitabine hydrochloride (GEM·HCl, C_9_H_11_F_2_N_3_O_4_·HCl) (MW = 299.66 g/mol), TPGS (MW = 1517 g/mol), Triton-X-100, bovine serum albumin (BSA), 1,2-dioleoyl-sn-glycero-3-phosphoethanolamine labeled with Atto 488 (DOPE-Atto 488), and RPMI-1640 medium supplemented with 10% of FBS (1% antibiotics addition) were acquired from Sigma-Aldrich (St. Louis, MO). Vitamin E succinate (MW = 530.80 g/mol) was bought from Santa Cruz Biotechnology Inc., (Dallas, TX). Hydrophilic polytetrafluoroethylene (PTFE) syringe filter (13 mm, 0.2 µm), hydrophilic PTFE syringe filter (25 mm, 0.4 µm), tris hydrochloride (Tris–HCl), and magnesium chloride (MgCl_2_) were obtained from Scharlab S.L. (Barcelona, Spain). EDTA-free Pierce Protease Inhibitor Tablets 4′,6-diamidino-2-phenylindole (DAPI) and Nile red were purchased from Thermo Fisher™ (Madrid, Spain). Materials and methods regarding VES–GEM chemical synthesis were described in the previous article (Pereira-Silva, Miranda-Pastoriza, et al. 2024). All additional reagents were of analytical or HPLC grade.

### 2.2. HPLC quantification method

VES–GEM content was quantified through high-performance liquid chromatography (HPLC) using RP-HPLC system JASCO (AS-4150 RHPLC Autosampler, PU-4180 RHPLC Pump, LC-Net II/ADC Interface Box, CO-4060 Column Oven, MD-4010 Photo Diode Array Detector), Zorbax Eclipse XDB-C18 column (5 μm, 4.6 mm × 250 mm) using methanol as mobile phase, flow rate 1 mL/min, 30 °C, 20 µL injection volume, and detection wavelength 248 nm (Fang et al. 2015). The HPLC calibration curve was constructed over a concentration range of 1–50 ppm, yielding a correlation coefficient (*R*^2^) exceeding 0.99. From this curve, the limit of detection (LOD) and limit of quantification (LOQ) were calculated to be 0.15 ppm and 0.236 ppm, respectively. Samples were adequately filtered before each injection using PTFE hydrophilic syringe filter (13 mm, 0.22 µm).

### 2.3. TPGS/VES–GEM micelles preparation

A set of TPGS/VES–GEM micelles was prepared varying TPGS-to-conjugate molar ratio, with fixed VES–GEM conjugate concentration, recurring to a solvent evaporation method (Table 1) (Grimaudo et al. 2018). First, a set of VES–GEM conjugate (1.33 mg) solutions in ethanol (1.67 mL) was prepared under stirring at 600 rpm (Cimarec i Poly 15; Thermo Scientific™, Fisher Scientific S.L., Madrid, Spain), room temperature (RT), protected from light, during 3 h. In parallel, TPGS micelle dispersions were prepared in PBS diluted in water (50:50 v/v, 5 mL) using different quantities of TPGS (1.95 mg, 3.9 mg, 7.8 mg, 15.6 mg, and 31.12 mg corresponding to 0.75/1, 1.5/1, 3/1, 6/1, and 9/1 polymer-to-conjugate molar ratio, respectively) and the mixture was kept stirring at 300 rpm till full dissolution of TPGS. When completely dissolved, VES–GEM conjugate solutions were added drop-by-drop to TPGS dispersions under gentle stirring (250 rpm). The system was maintained in a fume hood with magnetic stirring (250 rpm) at RT and protected from light to enable ethanol evaporation overnight (16 h) and assembly of TPGS/VES–GEM micelles. Blank TPGS micelles were prepared by direct dissolution accordingly, but without VES–GEM addition, and in PBS diluted in water (50:50 v/v, 5 mL).

**Table 1.** Composition of TPGS/VES–GEM micelles.

Polymer-to-conjugate molar ratio	TPGS (%w/v)	VES–GEM (%w/v)	VES–GEM in TPGS (%w/v)	Concentration TPGS (mg/mL)	Concentration TPGS (mM)	Concentration VES–GEM (mg/mL)
0.75/1	0.039	0.027	68.48	0.39	0.25	0.267
1.5/1	0.078		34.24	0.78	0.51	
3/1	0.156		17.12	1.56	1.03	
6/1	0.312		8.56	3.12	2.06	

For purification testing of TPGS/VES–GEM (6/1) micelles by filtration, the formulation was filtered through a hydrophilic PTFE syringe filter (25 mm, 0.4 µm), and VES–GEM content was calculated after aliquots dilution in ethanol 1:9 v/v. The influence of centrifugation (centrifuge model 5804R, Eppendorf AG, Hamburg, Germany) on TPGS/VES–GEM (6/1) micelles was tested in two parallel settings: centrifugation at 4000 rpm for 30 min and centrifugation at 12,000 rpm for 20 min, both at 4 °C (Grimaudo et al. 2018; Alambiaga-Caravaca et al. 2020). Collected supernatant aliquots were diluted in ethanol 1:4 v/v and VES–GEM content was analyzed through HPLC as described before. To assess the influence of ultrafiltration on TPGS/VES–GEM (6/1) micelles, Amicon Ultra 0.5 mL Centrifugal filters, 100 kDa (Merck Millipore, Carrigtwohill, Ireland) were used and aliquots of formulation were loaded and subjected to 4000 rpm, 4 min, at 25 °C (Rouco et al. 2022), the subnatant was collected and diluted 1:4 v/v in ethanol, and VESGEM content was determined through HPLC (Section 2.5.2).

### 2.4. Influence of VES–GEM conjugate on TPGS micellization process

The potential impact of VES–GEM on the self-assembly of TPGS micelles (CMC 0.132 mM, 0.2 g/L) was investigated using the surface tension test, which is a variation of the Du Noüy ring method. Briefly, a set of TPGS dispersions in PBS:water 50:50 v/v were prepared with concentrations of 0.025, 0.05, 0.25, 0.5, 2.5, and 5 mg/mL TPGS. To obtain a final volume of 5 mL, aliquots of ethanolic solution of VES–GEM conjugate (50 µL, 10.23 mg/mL) were added to glass vials containing the TPGS dispersions, final volume 5 mL. Controls were prepared accordingly, without VES–GEM addition. Subsequently, the formulations were allowed to equilibrate in a hood at RT and to permit ethanol evaporation; the latter was necessary for the micelles containing VES–GEM conjugates. Next, the surface tension of each solution (5 mL) was measured using a platinum ring in a tensiometer TD 1 Lauda (Fisher Scientific Hucoa, Madrid, Spain) (Liu et al. 2022).

### 2.5. TPGS/VES–GEM micelles characterization

#### 2.5.1. Micelle size, PDI, and zeta potential

The as-prepared TPGS/VES–GEM micelles (0.75/1, 1.5/1, 3/1, 6/1 and 9/1 molar ratio) were characterized regarding size, PDI, and zeta potential (ZP) by dynamic light scattering (DLS) (Zetasizer Nano ZS, Malvern Instruments, Malvern, UK). The measurement took place at 25 °C, samples without dilution and without filtration, in triplicates. For testing the influence of centrifugation on purification of TPGS/VES–GEM (6/1) micelle formulations, a parallel set of experiments was carried out by preparing 1.5 mL of formulation Eppendorf^®^ tubes further centrifuged at 4000 rpm, 30 min, 25 °C or 12,000 rpm, 20 min, 25 °C (Eppendorf^®^ 5804R, Hamburg, Germany) and size, ZP and PDI of the supernatant were measured through DLS in absence of filtration, in triplicates.

#### 2.5.2. Encapsulation efficiency and drug loading

Encapsulation efficiency (EE) and drug loading (DL) of VES–GEM conjugate in TPGS/VES–GEM (6/1) micelles was assessed by RP-HPLC system JASCO. Aliquots of the micelle formulation (100 µL) were diluted in ethanol (1:9 v/v) to allow micelle structure disruption, then vortexed for two seconds (Reax top model, Heidolph Instruments GmbH & Co. KG, Schwabach, Germany). The PTFE hydrophilic Scharlau syringe filter (13 mm, 0.22 µm) was used to filter the mixture, and HPLC quantification was performed afterward. EE (%) was computed as the percentage quotient of the quantified and initial weighted amounts of VES–GEM added to the mixture (Equation (1)), and DL (%) as the quotient, in percentage, between amount of quantified VES–GEM and combined weighted amount of VES–GEM and TPGS used to prepare the micelles (Equation (2)).

(1) EE(%)=quantified amount of VES−GEM conjugates in the micelles (mg)weighed amount of VES−GEM conjugates in TPGS micelles (mg)×100

(2) DL(%)=quantified amount of VES−GEM conjugates in the micelles (mg)weighed amount of VES−GEM conjugates+TPGS in the micelles (mg)×100 

#### 2.5.3. Transmission electron microscopy (TEM)

For morphology analysis, a set of blank TPGS and TPGS/VES–GEM (6/1) micelles were prepared in PBS:water (50:50 v/v), and negatively stained with 2% (w/v) phosphotungstic acid (2 min, RT), or uranyl acetate 2% (w/v) (5 min, RT) and viewed on a TEM JEOL JEM1011 at 80 kV (JEOL, Peabody, MA). Additionally, the morphology of the formulations stained with uranyl acetate was analyzed using FESEM (GeminiSEM, GEMINI 500, Zeiss, Oberkochen, Germany) 20 kV.

#### 2.5.4. UV–vis spectroscopy

Ethanolic solution of VES–GEM conjugate (0.267 mg/mL), TPGS micelles at 3.12 mg/mL loaded or not with VES–GEM conjugate (0.267 mg/mL), prepared in PBS:water 50:50 v/v, as well as diluted 1:9 v/v aliquots were analyzed through UV–vis spectroscopy. For dilution, VES–GEM conjugate was diluted in ethanol, and TPGS and TPGS/VES–GEM (6/1) micelles were diluted in PBS:water 50:50 v/v. UV spectra were recorded in the range 190–800 nm using an UV-Vis spectrophotometer Agilent 8534 (Waldbronn, Germany) (quartz cells with 10 mm light path). Blanks were ethanol for VES–GEM conjugate and PBS:water 50:50 for TPGS micelles.

### 2.6. Saturation solubility studies

To assess solubility of VES–GEM conjugate in TPGS micelles, a stock solution of VES–GEM in ethanol was prepared (5 mL, 0.8 mg/mL). Then, a set of aqueous TPGS dispersions (50:50 v/v PBS:water, 1 mL) with fixed polymer concentration (3.12 mg/mL) was prepared followed by addition of increasing concentrations of VES–GEM ethanolic solution, under stirring (300 rpm) and left overnight at RT, shielded from light, in a hood, to allow ethanol to evaporate (Table 2).

**Table 2.** Saturation solubility studies of VES–GEM in TPGS micelles.

TPGS concentration	Volume of TPGS dispersions (mL)	VESGEM added	Volume EtOH added
3.12 mg/mL	1	0.1 mg	0.125 mL
		0.2 mg	0.250 mL
		0.3 mg	0.375 mL
		0.4 mg	0.500 mL
		0.5 mg	0.625 mL
–		0.2 mg	0.250 mL

After ethanol evaporation, the formulations were subjected to centrifugation at 4000 × *g* for 30 min/4 °C (centrifuge model 5804R, Eppendorf AG, Hamburg, Germany) to eliminate insoluble VES–GEM aggregates. Then, the supernatants were collected and diluted in ethanol (1:4 v/v) followed by VES–GEM quantification by HPLC (Varela-Garcia et al. 2018; Alambiaga-Caravaca et al. 2020; Vivero-Lopez et al. 2022). Control was prepared as above, but without surfactant (VES–GEM, 0.2 mg, 0.8 mg/mL).

### 2.7. VES–GEM conjugate release profile

The dialysis method was used to evaluate the VES–GEM conjugate release profile, and HPLC was used to quantify the amounts released at pre-established intervals. Briefly, TPGS/VES–GEM (6/1) micelles were prepared in PBS:water 50:50 v/v (VES–GEM concentration 0.267 mg/mL) and 1 mL of formulation was transferred into dialysis membrane bags (MWCO 12.4 kDa; D9652-100FT, Sigma-Aldrich, St. Louis, MO) pretreated according to the manufacturer’s instructions. The dialysis bags were sealed and put into cups with release media (PBS:ethanol, 80:20 v/v, 15 mL) of pH 5.0 and 7.4 [24, 44] and incubated at 180 rpm and 37 °C (Di et al. 2017). Release media was sampled (0.25 mL) taken at predefined intervals (0.5, 1, 2, 4, 8, 24, 48, and 72 h) and the volume was replaced with new medium. The samples were diluted in ethanol (1:1 v/v) and filtered (0.22 μm membrane filter), and VES–GEM was quantified. The pH of release medium was monitored throughout duration of the drug release experiment to confirm pH values remained stable. The VES–GEM content inside the bags after seven days of release was further analyzed by diluting aliquots retrieved from the bag leftover followed by 1:9 v/v dilution in ethanol.

### 2.8. Stability of TPGS/VES–GEM micelles

(1) Stability testing for TPGS/VES–GEM (6/1) micelles prepared in PBS:water 50:50 v/v was carried out in accordance with previous studies, and at certain time points (day 0, 2, 7, and 28), both at 4 °C and 37 °C, and VES–GEM content was quantified through HPLC, the pH was recorded, and micelle size, ZP and PDI evaluated through DLS (Wang et al. 2014); (2) in protein corona-mimicking conditions by incubating TPGS blank micelles (3.12 mg/mL, 0.1 mL) with BSA (0.9 mL, 8 mg/mL), 1:9 v/v, further incubated at 100 rpm for 24 h at RT and followed by size, ZP, and PDI evaluation (Luo et al. 2022; He et al. 2023); (3) upon dilution, in which blank and TPGS/VES–GEM (6/1) micelles were diluted 1000 times in PBS:water 50:50 v/v and the size, ZP, and PDI changes compared by DLS (Grimaudo et al. 2018; Vivero-Lopez et al. 2022); (4) batch-to-batch variation, in which TPGS/VES–GEM micelles (3.12 mg/mL TPGS and 0.267 mg/mL VES–GEM, 3.75 mL) were independently prepared and characterized regarding size, size distribution by intensity, size distribution by number, and PDI.

### 2.9. BxPC3 cell membrane extraction and characterization

Commercially available BxPC3 cells (ATCC CRL-1687™, American Type Culture Collection, Manassas, VA) were cultured in RPMI-1640 medium supplemented with 10% FBS and 1% antibiotics (10,000 U/mL penicillin and 10,000 µg/mL streptomycin) in an atmosphere of 5% CO_2_ and 95% relative humidity at 37 °C, and collected (at 90% confluency), washed and trypsinized (10 min/37 °C) and fresh medium was added to neutralize trypsin action. The cell suspension was centrifuged (1200 × *g*/3 min/25 °C, Centrifuge 5417R Eppendorf AG, Hamburg, Germany). The resulting pellet was resuspended and washed with PBS buffer and centrifuged at 1200 × *g*/3 min. For hypotonic lysis, the cell pellet was resuspended in 1 mL hypotonic lysing buffer composed of 10 mM Tris·HCl, 10 mM MgCl_2_, and 1× EDTA-free protease inhibitor (Cao et al. 2021; Zou et al. 2023) and the mixture was incubated overnight protected from light in ice with shaking (190 rpm) (Incubator 1000, Heidolph Instruments GmbH & Co. KG, Schwabach, Germany). Later, the mixture composed of partially lysed cells was sonicated in ice container, to further disrupt cell structure, using Sonifier 450 ultrasonicator (Branson Ultrasonics, Branson Digital Sonifier Model 450, Danbury, CT) at 20% intensity, for 10 cycles of 5 s on/3 s off. Membrane fragments were isolated using a differential centrifugation protocol. Initially, cells were centrifuged at 3200 × *g* for 5 min at 4 °C. The supernatant was collected and subjected to a second centrifugation at 7000 × *g* for 10 min at 4 °C. The resultant supernatant was then centrifuged at 15,000 × *g* for 60 min at 4 °C. The pellet containing BxPC3 cell membrane fragments was washed, resuspended in 0.5 mL PBS, and stored at 4 °C. The genetic material content in the isolated membrane fragments was assessed using the Quant-iT PicoGreen dsDNA assay kit (Thermo Fisher, Waltham, MA), while the protein content was quantified using the Micro BCA™ protein assay kit.

### 2.10. Preparation and characterization of BxPC3 cell membrane-coated TPGS/VES

**Figure 2.**
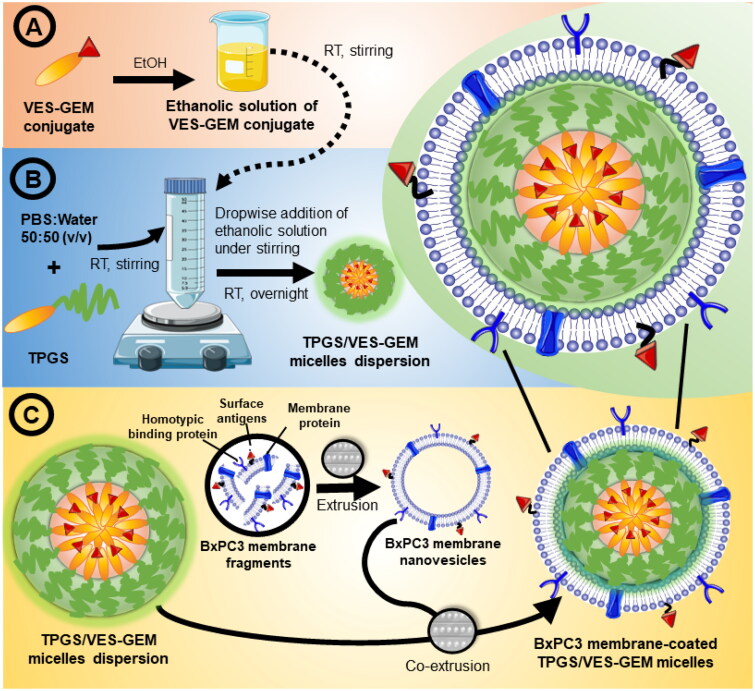
Preparation of BxPC3 cell membrane-coated TPGS/VES–GEM micelles through co-extrusion of BxPC3 membrane nanovesicles with TPGS/VES–GEM micelle cores. TPGS/VES–GEM micelles were prepared by dissolving VES–GEM in ethanol (A), and dropwise addition to TPGS aqueous dispersion (B). After solvent evaporation, BxPC3 cell membrane nanovesicles prepared through extrusion were added to the micelle formulation and co-extruded (200 nm pore size) to produce PC cell membrane-coated TPGS/VES–GEM micelles (C).

TPGS/VES–GEM (6/1) micelles were prepared by solvent evaporation method as described in previous sections (Figure 2(A,B)). BxPC3 cell membrane nanovesicles were prepared by diluting in water 1:4 v/v 0.72 mg/mL (0.086 mL, 0.06 mg of protein content) of membrane fragments in PBS, extruded using Avanti^®^ Mini Extruder (Avanti^®^ Polar Lipids, Birmingham, AL) 10 cycles (membrane pore 400 nm), and then 10 cycles (membrane pore 200 nm). TPGS/VES–GEM (6/1) micelles formulation was diluted five times with water and 0.2 mL (TPGS concentration 0.6 mg/mL) was co-extruded with the obtained BxPC3 membrane nanovesicles (2:1 polymer-to-membrane protein ratio) using membrane pore 200 nm for 10 cycles, for assembling BxPC3 cell membrane-coated TPGS/VES–GEM micelles (Figure 2(C)) (Pereira-Silva, Diaz-Gomez, et al. 2024). Control was a mixture of BxPC3 membrane nanovesicles with TPGS/VES–GEM micelles under the same concentrations, but not extruded. A separate set of experiments with new membrane batch was conducted to enhance the membrane protein-to-polymer ratio to 1:1 and to test the influence of extrusion on the process (Pereira-Silva, Diaz-Gomez, et al. 2024). For that, TPGS/VES–GEM (0.6 mg/mL polymer, 0.2 mL) was co-extruded with membrane fragments (0.72 mg/mL, 0.2 mL) under the same conditions to yield BxPC3 cell membrane-coated TPGS/VES–GEM micelles (1:1 polymer-to-protein ratio) and compared to the previously obtained system (2:1 w/w ratio). As control, TPGS/VES–GEM micelles (0.6 mg/mL), diluted 1:1 (v/v) in water were extruded with membrane pore 400 nm and 200 nm (10 cycles each) and added to 0.2 mL of BxPC3 nanovesicles (0.72 mg/mL, 0.2 mL). To assess if ultrasonication could be an alternative method for coating TPGS cores, a mixture of TPGS/VES–GEM (0.6 mg/mL polymer, 0.2 mL) and BxPC3 nanovesicles (0.2 mL, 0.06 mg of protein content), 2:1 w/w, was sonicated using Sonifier 450 ultrasonicator (Branson Ultrasonics, Branson Digital Sonifier Model 450, Danbury, CT) at 10% intensity, for 10 cycles of 3 s on/3 s off. The obtained structures were characterized regarding size, ZP, PDI, TEM, and confocal laser scanning microscopy (CLSM), as described previously.

### 2.11. Cell viability in 2D model

Cell viability was assessed in human PC cells (BxPC3 cell line, ATCC CRL-1687™, American Type Culture Collection, Manassas, VA). BxPC3 cells were cultured in RPMI-1640 medium supplemented with 10% FBS and 1% antibiotics (10,000 U/mL penicillin and 10,000 µg/mL streptomycin) in an atmosphere of 5% CO_2_ and 95% relative humidity at 37 °C. The ability to decrease cell viability of free GEM and VES–GEM, blank TPGS micelles, and TPGS/VES–GEM micelles was assessed using an AlamarBlue assay (ThermoFisher, Waltham, MA) by seeding 96-well plate (2 × 10^4^ cells/well) followed by incubating the cells for 24 h at 37 °C under the same conditions. Then, free GEM and VES–GEM, TPGS micelles, and TPGS/VES–GEM micelles (as prepared in Section 2.3) diluted in cell culture medium were added to the cells (*n* = 4), in various concentrations (100, 10, 1, 0.1, or 0.01 µM of VES–GEM conjugate or equivalent) followed by incubation for 72 h. BxPC3 cell membrane-modified TPGS/VES–GEM micelles (1:1 polymer-to-protein ratio, w/w) prepared as explained in Section 2.10 were tested accordingly, following a range of concentrations of 10, 1, 0.1, or 0.01 µM of VES–GEM conjugate or equivalent. GEM and TPGS micelles (blank and VES–GEM loaded) were prepared in PBS medium, and VES–GEM conjugate in DMSO. Cells cultured in culture medium or 2% DMSO solution in culture medium were used as negative controls. After incubation, culture medium was discarded, cells were washed twice with PBS and incubated with AlamarBlue reagent mixture (150 µL, 1:10 dilution in culture medium) following manufacturer’s protocol. The cells were incubated at 37 °C for 2 h and fluorescence of the supernatants was measured using a microplate FLUOstar Omega (BMG LABTECH, Ortenberg, Germany), at an excitation wavelength of 540 nm and emission wavelength of 580 nm and cell viability was calculated (Equation (4)):

(4) $$\text{Cell viability }(\%)=\frac{{\text{RFU}}_{\text{exp}\;}-{\text{RFU}}_{\text{blank}}}{{\text{RFU}}_{\text{control}\;}-{\text{RFU}}_{\text{blank}}}\times 100\;$$

*RFU_exp_* stands for sample relative fluorescence units, *RFU_blank_* is the blank relative fluorescence units, and *RFU_control_* is the control relative fluorescence units (AlamarBlue reaction in non-treated cells). For IC_50_ calculation and obtainment of respective curves, GraphPad^®^ Prism^®^ software (version 10.1.2; San Diego, CA) was used.

### 2.12. Cell viability in 3D model

A 3D *in vitro* model of PC was utilized to assess the effectiveness of each formulation by using collagen hydrogels containing BxPC3 cells for replicating the desmoplastic microenvironment of PC (Liu, Xu, et al. 2016; Wang et al. 2016). The concentration of collagen type I (Col1) was determined using micro BCA (Thermo Fisher Scientific, Waltham, MA) after it was extracted from rat tail tendons (Hou et al. 2019; Yang et al. 2020). The collagen dispersion was neutralized with NaOH 1 M and DPBS 10× was added to adjust the osmolarity of the dispersion, to which cell suspension and whole cell media were combined to get a final BxPC3 cell density of 3 × 10^6^ cells/mL and a final Col1 concentration of 4 mg/mL. The pre-gels were incubated for 30 min at 37 °C (25 μL/well) and 300 μL of cell medium was carefully added to each well and the hydrogels cultured for four days. Cell medium was replenished every other day. Then, medium was collected and AlamarBlue was used to assess the effectiveness of VES–GEM and the TPGS formulations against the cell-laden hydrogel 3D model, by adding each formulation (200 µL) to the wells followed by an incubation period of 72 hours (*n* = 4) and following the protocol set in Section 2.12. AlamarBlue was used to assess the cell viability of the tumoroids both before and after treatment for metabolic activity normalization. Untreated cell-laden hydrogels were negative controls (non-treated or treated with DMSO 2%).

### 2.13. Cell uptake of TPGS/VES–GEM micelles

For cellular uptake studies, BxPC3 cells were cultured as described previously (Section 2.9) and seeded on eight-well glass slides (Lab-Tek II chamber slides; Thermo Scientific, Waltham, MA) at a density of 5 × 10^4^ cells/well and incubated overnight. Then, cells were incubated with PBS-diluted Nile red-loaded TPGS/VES–GEM micelles (0.9 mg/mL TPGS, correspondent 100 µM concentration point, 2.9 µg/mL Nile red) for 1 h (Pereira-Silva, Diaz-Gomez, et al. 2024). Control blank TPGS micelles and Nile red-loaded TPGS micelles were prepared as described previously in Section 2.3, in the latter case Nile red replacing VES–GEM (TPGS concentration 3.12 mg/mL, 10 µg/mL Nile red concentration). All micelles were filtered through a hydrophilic PTFE syringe filter (25 mm, 0.4 µm) to remove any unincorporated Nile red. BxPC3 cells were then washed with PBS three times, fixed with 4% paraformaldehyde (100 µL/well) during 10 min and washed again thrice with PBS, followed by incubating with Triton X-100 (0.2% dilution in PBS) for 5 min, and rinsed thrice with PBS (Pereira-Silva, Diaz-Gomez, et al. 2024). Then, cells were dyed with DAPI, adequately covered with a glass coverslip and observed through CLSM recurring to Stellaris 8 confocal microscope (Leica Microsystems; Wetzlar, Germany).

### 2.14. Statistical analysis

The statistical data analysis was performed with version 10.2 of the GraphPad^®^ Prism^®^ program (San Diego, CA). When appropriate, the mean ± standard deviation was used to express each outcome. A two-way ANOVA was used to analyze the *in vitro* results, and when necessary, the post hoc Tukey honest significance test was performed afterward. Values lower than 0.05 were considered for statistical significance.

## 3. Results and discussion

### 3.1. Preparation and characterization of TPGS/VES–GEM micelles

TPGS, a soluble VE derivative, was chosen as amphiphilic building block for encapsulating VES–GEM on account of its ability to solubilize with good efficiency poorly water-soluble drugs and enable higher drug loading when compared to other surfactant with larger molecular weights. Second, TPGS has been reported to self-assemble into small micelles (<50 nm), which could be favorable to facilitate penetration in solid tumors bearing dense stroma network, such as PC (Cabral et al. 2011). Improved stroma permeation leads to improved accumulation and accessibility to cancer cells which can leverage therapeutic outcomes of the payload that is delivered through the nanosystem. Third, the PEGylated surface can potentially prolong biologic half-life of the loaded drug and ameliorate pharmacokinetics. Lastly, the intrinsic biological features ranging from anticancer activity and antioxidant activity endow TPGS with additional exciting attributes as a functional excipient and is therefore an attractive excipient for micelle development. VES is a bioactive compound integrating TPGS structure and has been explored as building block for development of VES-drug conjugates (Duhem et al. 2014; Liang and Qiu 2021; Pereira-Silva, Diaz-Gomez, et al. 2024) and also integrating the structure of nanosystems to promote self-assembly, structural stability, drug solubility, and bioactive properties (Yan et al. 2024), such as hydrophobic modification of poloxamer micelles (Liu, Xu, et al. 2016), modification of hyaluronic acid micelles (Wang et al. 2016; Hou et al. 2019; Yang et al. 2020), construction of VES-g-polylysine micelles (Xu et al. 2017), modification of chitosan (VES-g-chitosan) in TPGS micelles (Chen et al. 2017; Wu et al. 2017; Yuan et al. 2018), and poly(2-ethyl-2-oxazoline)–VES micelles (Qu et al. 2018).

**Figure 3.**
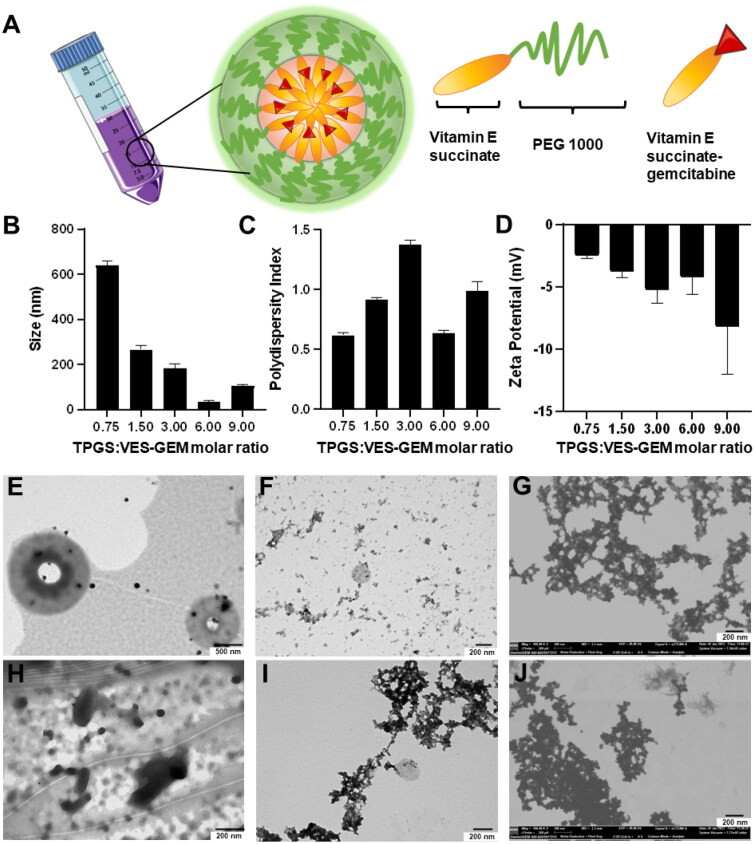
(A) Schematic illustration of TPGS/VES–GEM micelles. Size (intensity) (B), PDI (C), ZP (D) and of TPGS/VES–GEM conjugates micelles prepared with 0.75/1, 1.5/1, 3/1, 6/1, and 9/1 TPGS-to-VES–GEM molar ratios in PBS:water medium (50:50 v/v). TEM micrographs of blank TPGS micelles stained with phosphotungstic acid 2% w/v (E), uranyl acetate 2% w/v (F), and FESEM pictures of the same formulation dyed with uranyl acetate 2% w/v (G). TEM micrographs of TPGS/VES–GEM (6/1) micelles stained with phosphotungstic acid 2% w/v (H), uranyl acetate 2% w/v (I), and FESEM micrographs of the loaded micelles dyed with uranyl acetate 2% w/v (J).

TPGS/VES–GEM micelles were prepared recurring to a solvent evaporation method due to its applicability and simplicity (Figure 3(A)). Ethanol was left evaporating overnight (16 h) under stirring with airflow to allow removal of ethanol and slow self-assembly of the nanocarrier by arrangement of VES–GEM and building blocks. TPGS/VES–GEM micelles bearing increasing polymer-to-VES–GEM 0.75/1, 1.5/1, 3/1, 6/1, and 9/1 molar ratios were prepared, and TPGS/VES–GEM (6/1) showed the lowest size (∼30 nm), PDI ∼0.5 and slightly negative surface charge (∼−4 mV) (Figure 3(B–D)). A mixture of PBS:water 50:50 v/v was selected as preparation medium to compensate for the intrinsic osmotic pressure of the micelle system and maintain an equilibrated ionic microenvironment for improved micelle dispersion stability.

TEM and FESEM pictures of blank TPGS (3.12 mg/mL) (Figure 3(E–G)) and TPGS/VES–GEM (6/1) micelles (3.12 mg/mL, VES–GEM at 0.267 mg/mL) (Figure 3(H–J)) showed presence of spherical structures and large agglomerates. For TPGS/VES–GEM (6/1) micelles prepared in PBS:water (50:50 v/v), the EE was very high (96.55 ± 3.84%) and the drug loading was substantial (7.63 ± 0.28%) as TPGS is suggested to pose as good solubilizer with low molecular weight. The high PDI values observed for the different ratios are in agreement with TEM pictures and indicate presence of highly heterogeneous population, this is, co-existence of multiple particle populations, including large aggregates. This result is likely due to either the self-assembly dynamics of TPGS, insufficient solubilization of TPGS, the hydrophobic nature of the VES–GEM conjugate, and the absence of a purification step, which may allow unincorporated material (polymeric or drug-based) or associated aggregates to prevail. Additionally, the micelles may tend to aggregate due to the absence of strongly charged surface, hence several studies recur to filtration of TPGS micelles to remove larger aggregates. The addition of co-surfactant is also another strategy often used to improve stability and drug solubilization capacity of TPGS systems.

**Figure 4.**
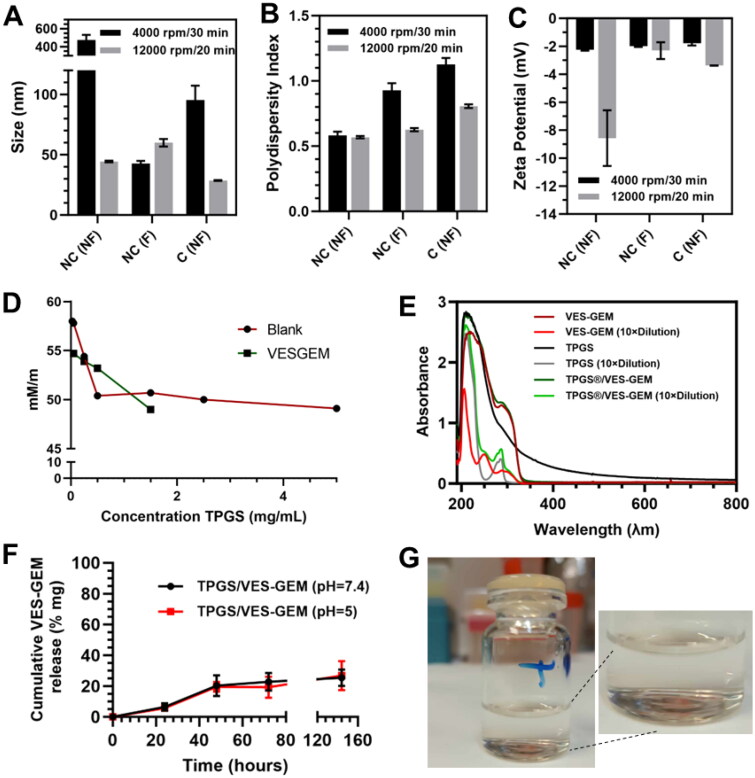
Size (A), PDI (B), and ZP (C) of two distinct batches of TPGS/VES–GEM (6/1) micelles without filtration, after filtration (0.4 µM filter) and after centrifugation at 4000 rpm/30 min/25 °C for the first batch – black colored bar – or 12,000 rpm/20 min/25 °C for the second batch – grey-colored bar. (D) CMC determination through surface tension measurement (mM/m) at 25 °C ± 0.5 °C, using *Du Noüy* ring method. (E) UV–vis absorption spectra of VES–GEM (0.267 mg/mL and 0.0267 mg/mL) in ethanol, TPGS (3.12 mg/mL and 0.312 mg/mL of polymer), and TPGS/VES–GEM (6/1) micelles (3.12 mg/mL of polymer, 0.267 mg/mL VES–GEM, and 0.312 mg/mL of polymer, 0.0267 mg/mL VES–GEM) in PBS:water 50:50 v/v, RT. (F) Drug release patterns for TPGS/VES–GEM (6/1) micelles at pH 5 and pH 7.4. The micelles were incubated at 37 °C (100 rpm) and the release medium was PBS supplemented with ethanol (80:20 v/v). (G) Physical appearance of TPGS/VES–GEM (6/1) micelle dispersion. NC: non-centrifuged; NF: non-filtered; C: centrifuged; F: filtered.

Next, filtration effect (0.4 µM filter pore) and centrifugation effect on size, PDI, ZP, and EE of VES–GEM in TPGS micelles was explored. Filtration of TPGS/VES–GEM micelles resulted in a significant reduction in particle size, as illustrated in Figure 4(A). This reduction may be attributed to the removal of aggregates, which could explain the observed variability. Typically, the micelle population exhibits an average size of approximately 40 nm, as seen in the sample subjected to centrifugation at 12,000 rpm. Filtration of such small size population did not appear to affect the size and the PDI (Figure 4(B,C)). This is likely due to the substantial difference between the pore size of the filter and the micelle system. In contrast, centrifugation reduced the particle size, likely by eliminating aggregates, and increased the PDI, possibly due to some degree of destabilization. Surface charge remained unaffected by either procedure (Figure 4(C)).

VES–GEM content in TPGS/VES–GEM (6/1) micelles showed variations before and after centrifugation at 12,000 rpm/20 min (Table 3), which may be attributed to removal of unloaded VES–GEM or to destabilization of the system. Initial EE, without purification, was approximately 100%, and the dispersions were transparent without any visible aggregates and precipitates, which may indicate successful loading. However, some non-visible agglomeration phenomena may be present, and the EE could be overestimated. It is unclear whether the maintenance >100% EE in the 4000 rpm centrifugation group is due to almost complete VES–GEM encapsulation or insufficient centrifugal forces to precipitate unloaded VES–GEM. In the case of more drastic centrifugal forces, the decrease in VES–GEM content may be attributed also to collapse of the structure, which renders this method of purification inconclusive.

**Table 3.** Encapsulation efficiency of VES–GEM conjugate in TPGS/VES–GEM (6/1) micelles after centrifugation, RT.

Formulation	Centrifugation setting	EE VES–GEM (EE ± S.D.%)	
		Non-centrifuged	Centrifuged
TPGS/VES–GEM (6/1)	4000 rpm/30 min	115.06 ± 1.19	102.97 ± 6.92
	12,000 rpm/20 min	115.53 ± 2.30	61.10 ± 1.50

Filtration of TPGS/VES–GEM (6/1) shows negligible decrease in VES–GEM content in the formulation (EE = 95.07 ± 5.15%), suggesting almost complete encapsulation, or presence of unloaded VES–GEM in structures sized <400 nm. The filter showed no apparent retention of VES–GEM nor micelles, which would decrease substantially VES–GEM quantified.

With the intent to estimate unloaded VES–GEM, TPGS/VES–GEM (6/1) were subjected to gentle ultrafiltration in order to collect the first drops which could provide an estimation for EE with enhanced accuracy (Table 4).

**Table 4.** Encapsulation efficiency of VES–GEM conjugate in TPGS/VES–GEM (6/1) micelles, after ultrafiltration with Amicon filter (0.5 mL, 100 kDa pore size).

Formulation	Concentration VES–GEM after filtration (µg/mL ± S.D.)	Ratio concentration VES–GEM after/before ultrafiltration (% ± S.D.)	EE VES–GEM (EE ± S.D. %)
TPGS/VES–GEM (6/1)	257.76 ± 0.02	99.99 ± 0.01	96.54 ± 0.01

Results showed almost total absence of VES–GEM in the subnatant, suggesting negligible unloaded VES–GEM for TPGS/VES–GEM (6/1), with TPGS concentration 3.12 mg/mL. Thus, the formulation was used for further studies without any purification step.

The CMC of TPGS has been reported to be 0.2 mg/mL and surface tension experiments evidenced a marked decrease in surface tension for TPGS 0.1–1 mg/mL concentration range. VES–GEM presence slightly increased CMC, possibly because some TPGS molecules were involved in VES–GEM solubilization and, therefore, less surfactant was available at the liquid–air interface (Figure 4(D)). UV–vis absorption peaks suggested encapsulation of VES–GEM in TPGS micelles (Figure 4(E)). TPGS/VES–GEM (6/1) formulation showed clear, limpid aspect (Figure 4(G)).

The formulation was able to display controlled release profile, achieving ∼30% cumulative VES–GEM release after seven days, with a similar profile at both pH, and with PBS medium supplemented with ethanol 80:20 v/v (Figure 4(G)). Inside the bag VES–GEM content was considerable, 0.187 ± 0.004 mg/mL and 0.237 ± 0.054 mg/mL for pH = 7.4 and pH = 5, respectively. Ethanol was employed as an alternative to surfactant supplementation (e.g. SDS, Tween 80) in order to improve solubility of VES–GEM in the receptor medium and enable sink conditions (Vivero-Lopez et al. 2022). Ethanol was chosen since it is one of the less toxic organic solvents in which VES–GEM displays high solubility. In the absence of ethanol, when the medium was PBS supplemented with Tween 80 0.5% v/v or Tween 80 1% v/v no appreciable release was verified after seven days. The slow and modest release at the conditions of the experiment can possibly suggest that, after seven days, most of the drug can still be in the core of the TPGS micelles, which can be mediated by a combination of strong hydrophobic interactions established between TPGS and VES–GEM (both bearing VES moieties) and also VES–GEM/VES–GEM. It shows the ability of the system to retain VES–GEM (and its active drug GEM) for long periods of time with high stability functioning as a drug reservoir toward long-action formulation (Gao et al. 2008). Nevertheless, it is expected that *in vivo* conditions may facilitate VES–GEM release, also taking into account the limitations in mimicking physiological fluids using PBS:ethanol 80:20 v/v as release medium.

Solubility studies were performed to quantify the VES–GEM solubilizing capacity of TPGS. Results elicited that, even for VES–GEM concentration twice the ones used in this work (0.5 mg/mL), no aggregate was formed. The quantified VES–GEM was near the theoretical one, suggesting TPGS capability to fully solubilize VES–GEM at surfactant concentrations of 3.12 mg/mL and VES–GEM concentrations of 0.267 mg/mL (Table 5).

**Table 5.** Saturation solubility studies of VES–GEM conjugate in TPGS micelles.

TPGS conc.	VES–GEM (mg)	Volume EtOH (mL)	Aspect after overnight evaporation	Aspect after centrifugation	Expected VESGEM content if < solubility (mg/mL)	VESGEM content (mg/mL) ± S.D.
3.12 mg/mL	0.1 mg	0.125	Transparent	Transparent	0.1	0.15 ± 0.006
	0.2 mg	0.25	Transparent	Transparent	0.2	0.25 ± 0.002
	0.3 mg	0.375	Transparent	Transparent	0.3	0.36 ± 0.002
	0.4 mg	0.5	Transparent	Transparent	0.4	0.56 ± 0.006
	0.5 mg	0.625	Transparent	Transparent	0.5	0.42 ± 0.021
–	0.2 mg	0.25	Aggregates (flakes)	Small precipitate	∼0	n.d.

Samples were analysed through HPLC after centrifugation 4,000 g/30 min, 4 ºC.

Samples were analyzed through HPLC after centrifugation 4000 × *g*/30 min, 4 °C.

### 3.2. Stability of TPGS/VES–GEM micelles

TPGS/VES–GEM (6/1) micelles in PBS:water (50:50 v/v) buffer maintained the pH near 7.4 (Figure S1(A)). Throughout the duration of the experiment, 30 days, the VES–GEM content in the formulation decreased gradually, showing a similar reduction after 30 days at both 4 °C and 37 °C. The formulations maintained a concentration of VES–GEM >200 ppm, which may indicate the micelles may help protecting the conjugate from extensive degradation (Figure S1(B)). Nonetheless, the formulations kept at 37 °C showed increased variations in VES–GEM content at intermediate time points which may be due to the action of temperature, to the stirring that the formulations were subjected (180 rpm) or to measurement errors.

Considering DLS measurement, the size of the micelles increased slightly through time, which may be due to aggregation (Figure S1(C)). PDI started at day 0 with ∼0.3 and was maintained almost constant, as opposed to the formulations kept at 4 °C, which had PDI doubling after 30 days (Figure S1(D)). This is probably due to the absence of agitation in the group of formulations kept at 4 °C, which triggers aggregation and precipitation phenomena. ZP results evidenced minimal variation in surface charge in both groups (Figure S1(E)). Overall, TPGS/VES–GEM (6/1) micelles showed stability in terms of size, PDI, surface charge, VES–GEM release profile and at both storage and physiological temperatures. No precipitate nor sediment was observed when the formulations were kept at RT for 1 week.

PEGylation offers steric hindrance and a hydrated shell at the surface of TPGS/VES–GEM micelles that is known to avoid uptake from immune cells and prolong blood circulation half-life of payload (Wang et al. 2014; Di et al. 2017; Wu et al. 2020; Han et al. 2022). Protein corona formation on PEGylated micelles has been underexplored and, while it has been suggested not to be negligible, it depends on several factors such as morphology, physicochemical properties and surface charge, in which positively charged structures may favor deposition of serum proteins through electrostatic interactions and adsorption and formation of a protein corona (Richtering et al. 2020; Cai et al. 2022; Wang et al. 2024). Regarding TPGS micelles, two recent studies have explored the influence of protein corona coating on *in vitro* and *in vivo* performance and have shown propensity to be coated by BSA and human serum albumin (HSA) (Qin et al. 2022; Wu et al. 2023). Still, considering the neutral or slightly negative surface charge and the PEG shell, TPGS/VES–GEM (6/1) micelles are not expected to be prone to be coated by serum proteins, such as albumin.

In our study, a simplified protein corona-mimicking composition was explored (BSA) to assess the potential impact of such protein coating (Figure S1(F–H)). The results were not conclusive, as a significant decrease in size from 30 nm to ∼10 nm, instead of a slight increase, may indicate the presence of a population of BSA nanoparticles in high intensity and number and influence the results. A control solution of BSA at the same concentration used in this study had particles of size ∼8 nm, which may consubstantiate this claim. Hence, either a BSA corona was formed at the surface of TPGS/VES–GEM micelles and could not be detected by DLS, or the formulation consisted of a mixture of non-coated TPGS/VES–GEM micelles with BSA nanoparticles. In either case, it could be expected a decrease in ZP, in the first one due to coating with the negatively charged protein BSA thus shifting to surface charge to more negative values, and in the second case, the presence of BSA population in high number could shift the ZP values to more negatively charged ones as reported in literature for albumin-based nanoparticles (Chen, Jia, et al. 2024).

Both blank and VES–GEM loaded TPGS micelles showed drastic changes in size after 1:1000 dilution (Table 6).

**Table 6.** Stability upon dilution of TPGS/VES–GEM (6/1) micelles (1:1000 dilution) after 1 h equilibrating/RT without stirring.

Formulation	Size (nm) ± S.D.	PDI ± S.D.	ZP (mV) ± S.D.
TPGS	737.07 ± 287.17	1.08 ± 0.19	−6.49 ± 6.48
TPGS/VES–GEM	234.51 ± 234.23	0.50 ± 0.25	−3.65 ± 0.75

**Figure 5.**
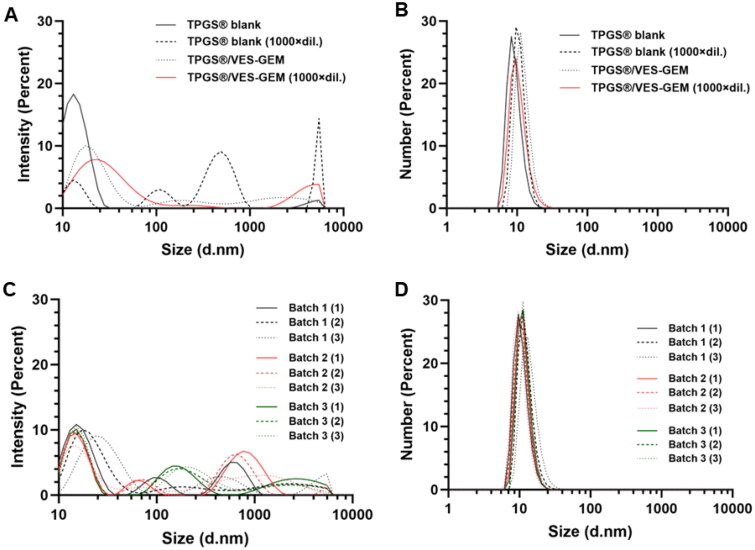
DLS size distribution, intensity mode (%) (A) and number mode (%) (B) of TPGS and TPGS/VES–GEM (6/1) micelles, prepared in PBS:water 50:50 v/v, non-diluted or diluted 1:1000 in PBS. Size distribution, intensity mode (%) (C) and number mode (%) (D) of three distinct batches, prepared in different days, in triplicates, of TPGS/VES–GEM (6/1) micelles, prepared in PBS:water 50:50 v/v.

As CMC of TPGS has been reported to be near 0.2 mg/mL, such high dilution yielded final TPGS concentration 100 times below CMC and the micelles may not be theoretically stable under such dilution regimen. It is possible some micelle-like structures can persist transiently below the CMC value due to some degree of kinetic stability and dynamic equilibrium. Some degree of rearrangement may also be at play here, in which the small micelles may suffer rearrangement and reorganize into bigger structures, also possibly forming VES–GEM aggregates. When the diluted formulations were analyzed regarding the size distribution by intensity, two peaks at higher intensities appeared for blank TPGS micelles diluted 1000 times, and one for the diluted TPGS/VES–GEM (6/1) micelles, the latter accompanied by flattened peak near 20 nm, which explain the change in size observed for the formulations (Figure 5(A)).

The same was not verified in size distribution in number (Figure 5(B)) which exhibited almost coincident peaks near size 10 nm. Nonetheless, the vast array of TPGS micelles developed so far have reached promising *in vivo* results which may suggest these structures are able to withstand – even only partially – physiological dilution, at least in mice models (Mahajan and Patil 2020; Li et al. 2021; Gai et al. 2023). Additional crosslinking procedures and addition of a co-surfactant may also help leveraging stability of TPGS micelles (Wang et al. 2019; Ding et al. 2020; Li et al. 2020; Wang et al. 2020; Zhou et al. 2022; Metwally et al. 2024). The shear stress resistance of the micelles may also be subject of further study at hemodynamic conditions, in order to ascertain the influence of the micelle core compactness on *in vivo* stability (Zhang et al. 2022). TPGS/VES–GEM (6/1) micelles did not show significant batch-to-batch variation as size kept ∼40 nm for the three independently prepared formulations (Table 7).

**Table 7.** Batch-to-batch variation of TPGS/VES–GEM (6/1) micelles (*n* = 3).

Formulation		Size (nm) ± S.D.	PDI ± S.D.
TPGS/VES–GEM	Batch 1	40.06 ± 8.16	0.461 ± 0.067
	Batch 2	36.12 ± 2.04	0.683 ± 0.034
	Batch 3	37.28 ± 3.65	0.605 ± 0.045

Additionally, the three independent batches the correspondent triplicates, showed almost coincident population distribution with similar peaks (Figure 5(C,D)).

### 3.3. Coating of TPGS/VES–GEM micelles with tumor cell membranes

**Figure 6.**
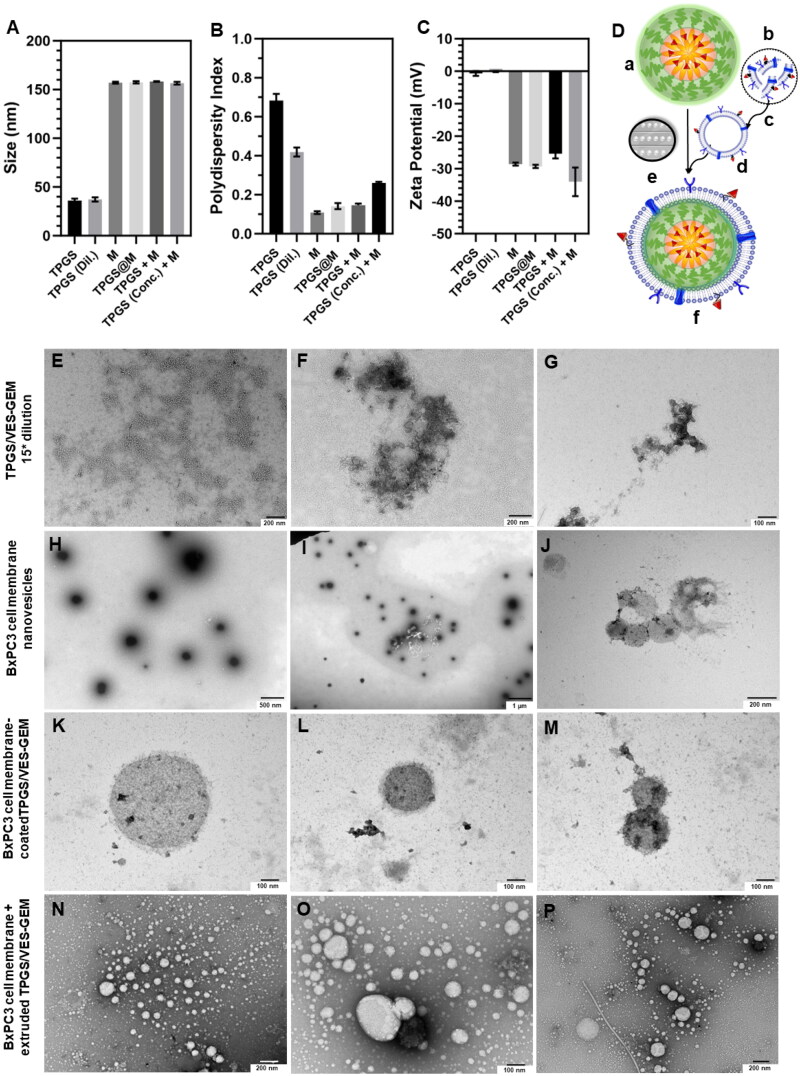
Size (A), PDI (B), and ZP (C) of TPGS/VES–GEM micelles (polymer concentration of 0.63 mg/mL, 1:5 dilution in water, TPGS), TPGS/VES–GEM micelles final dilution (polymer concentration of 0.22 mg/mL, TPGS (Dil.)), BxPC3 nanovesicles (M), BxPC3 membrane-coated TPGS/VES–GEM micelles (polymer-to-protein ratio of 2:1, TPGS@M), mixture of TPGS/VES–GEM micelles (polymer concentration of 0.63 mg/mL, 1:5 dilution in water) with BxPC3 nanovesicles and diluted for DLS measurement, TPGS + M, and a concentrated TPGS (3.12 mg/mL) with BxPC3 nanovesicles mixture. (D) Preparation of BxPC3 cell membrane-coated TPGS/VES–GEM micelles through co-extrusion of micelle cores with BxPC3 cell membrane nanovesicles: (a) TPGS/VES–GEM nanoparticle preparation by solvent evaporation; (b) extraction of BxPC3 membrane fragments; (c) extrusion of the BxPC3 membrane fragments; (d) assembly of BxPC3 cell membrane nanovesicles; (e) co-extrusion of TPGS/VES–GEM nanoparticles with BxPC3 nanovesicles and (f) fabrication of BxPC3 cell membrane-coated TPGS/VES–GEM micelles. TEM pictures of the TPGS/VES–GEM micelles 1:15 dilution in water (E–G), BxPC3 nanovesicles (H–J), BxPC3 cell membrane-coated TPGS/VES–GEM micelles (polymer-to-protein ratio of 1:1) (K–M), and mixture BxPC3 cell membrane nanovesicles with TPGS/VES–GEM micelles (N–P). Three photos were taken independently from the same prepared sample for each formulation. The samples were not filtered. Samples were stained with phosphotungstic acid 2%.

Inspired by the former results, a strategy was devised to explore the feasibility of coating TPGS/VES–GEM micelle system with a PC-derived biological membrane by co-extrusion of assembled BxPC3 nanovesicles with TPGS/VES–GEM micelles as cores. Typically, triblock copolymers like poloxamer 188 (Duan et al. 2022), poloxamer 407 (Jin et al. 2024; Ren et al. 2024; Zhang et al. 2024), and diblock copolymers like PEG-PCL (Malhotra et al. 2021; Gong et al. 2023) have received the most attention in regards to assembling cell membrane-coated polymeric micelles. The closest report would be coating – or encapsulation – of several TPGS micelles by a synthetic lipid bilayer (Li et al. 2024), and this was also applied to other micelle core composition (Wu et al. 2024). In addition to its strongly hydrophilic surface, neutral surface charge and dynamic nature of micelles, and their small size (<40 nm) are additional challenges for a successful coating. In this work, BxPC3 cell membrane nanovesicles were prepared through sequential extrusion and showed size ∼150 nm, low PDI and strongly negative surface charge (∼−30 mV). For coating TPGS/VES–GEM micelles, co-extrusion of the micelles with the previously formed BxPC3 nanovesicles (M) was pursued and the final coated structure – denoted as TPGS@M – showed similar size, ZP, and PDI to the BxPC3 nanovesicles (Figure 6(A–D)). This may indicate that, instead of a successful coating of TPGS cores, the micelle cores could be encapsulated in the pre-formed BxPC3 nanovesicles. In that case, a decrease in PDI was expected to occur, and not an increase, as the population ∼40 nm corresponding to the micelle cores would decrease. Moreover, the control group of mixture of BxPC3 nanovesicles with micelle cores (TPGS + M) showed similar size, PDI, and ZP, which supports the hypothesis that TPGS/VES–GEM micelles were not coated nor encapsulated, but are present in a mixture with BxPC3 nanovesicles, either unaltered or altered, the latter regarding probable intercalation of TPGS in BxPC3 lipid bilayer (Pereira-Silva, Diaz-Gomez, et al. 2024). The ZP observed for the coating formulation was similar to BxPC3 nanovesicles (∼−30 mV), which could indicate, even if partially, some degree of coating. Regarding TEM pictures, BxPC3 nanovesicles were clearly observed in the ‘M’ group (Figure 6(H–J)), as well as in subsequent TPGS@M (Figure 6(K,M)) and TPGS + M (Figure 6(N–P)) groups. However, no evidence of a coating or encapsulation was noticed. The quality of the micelle cores may also not be the most adequate as shown in Figure 6(E–G), in which the high PDI and agglomeration may hamper the coating process.

These results were also similar to the TEM pictures obtained by a more frequently employed staining consisting of uranyl acetate 2% w/v (Figure S2(A–L)). Overall, it seems coating of individualized TPGS micelles with cell membranes is not likely to occur taking into consideration DLS and TEM results. Other techniques may help identify any hypothetical biomembrane coating.

Next, CLSM was utilized to analyze the appearance of dye-labeled BxPC3 cell membrane-coated TPGS micelles. Figure S3 shows a structure ∼100 nm of green color suggestive of the DOPE-Atto-labeled bilayer. No red color was detected. These results corroborate DLS and TEM analysis regarding the presence of BxPC3 membrane nanovesicles and possible disintegration of TPGS structures, aided by dilution effect. A further attempt at fabricating BxPC3 cell membrane-coated TPGS/VES–GEM micelles was tried decreasing polymer-to-protein ratio to 1:1 w/w and by including a control of BxPC3 nanovesicle and extruded TPGS/VES–GEM micelles. The results were compared to the previously obtained ones. A similar pattern was observed for the coated group TPGS@M (1:1 w/w) which exhibited similar sizes and PDI to the 2:1 w/w counterparts (TPGS@M) and to the control mixtures (Figure S4(A–C)).

These results indicated that decreasing the polymer-to-protein ratio was not significant for assembling such coated structures and were not conclusive regarding possible encapsulation of TPGS micelles. Above all, the final formulation after coating has strongly negative surface charge (∼−20 mV) which is close to the parental BxPC3 membrane nanovesicles (∼−28 mV) and suggests the presence of cell membrane material-based structures, probably BxPC3 cell membrane nanovesicle population co-existing with TPGS/VES–GEM micelles and TPGS-modified BxPC3 nanovesicles.

Lastly, ultrasonication was also employed to produce BxPC3 cell membrane-coated micelles. Here, a mixture of BxPC3 nanovesicles and TPGS/VES–GEM micelles (1:4 v/v) was ultrasonicated to force rearrangement of nanovesicles around TPGS cores, at 2:1 w/w. The differences regarding size, ZP and PDI were more expressive, particularly size and PDI variations between BxPC3 nanovesicles (M), coated structures (TPGS@M (US)), and control group (TPGS(US) + M) (Figure S5(A–D)).

Notably, and for the first time, the control mixture of micelle cores and nanovesicles showed increased size when compared to the coated group (∼120 nm), which could elicit the formation of a new type of structure. TPGS@M(US) still display size considerably higher when compared to the cores, which is not suggestive of a coating. The distinct size also does not relate to a successful encapsulation of TPGS micelle cores which would also shift ZP values to the ones observed for nanovesicles and increase the ones obtained for the control mixture group. PDI values were slightly higher for the control mixture group when compared to the coated group, eliciting the presence of a more polydisperse population resulting from the mixture of two populations with significant size variations (∼40 nm for TPGS cores and ∼150 nm for BxPC3 nanovesicles). Above all, both ultrasonication and extrusion methods produced systems composed of BxPC3 cell membranes and TPGS/VES–GEM micelles with unclear structure and disposition, with sizes in the 120–150 nm range, PDI < 0.4, and strong negative surface charge (ZP < −20 mV), resembling the ones of native cell membrane structures (Cao et al. 2021; Zou et al. 2023).

### 3.4. *In vitro* cell studies in 2D and 3D models

**Figure 7.**
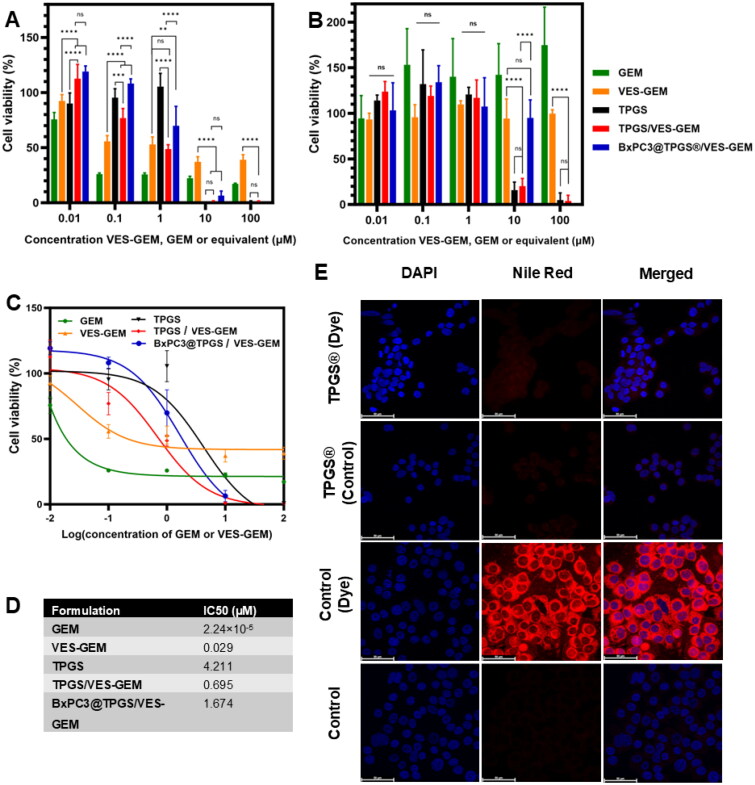
(A) *In vitro* cytotoxicity of GEM, VES–GEM, TPGS, TPGS/VES–GEM (6/1), and BxPC3 cell membrane-modified TPGS/VES–GEM micelles at various concentrations (0.01 μM, 0.1 μM, 1 μM, 10 μM, and 100 μM) and expressed as cell viability (%) using AlamarBlue assay kit. (B) *In vitro* cytotoxicity of VES–GEM, TPGS, TPGS/VES–GEM (6/1) micelles, and BxPC3 cell membrane-modified TPGS/VES–GEM micelles at various concentrations (0.01 μM, 0.1 μM, 1 μM, 10 μM, and 100 μM), in BxPC3/collagen hydrogel 3D model. Results expressed as cell viability (%) recurring to AlamarBlue assay kit. Dilutions were made in cell culture medium. For BxPC3 cell membrane-modified TPGS/VES–GEM micelle group concentration range was 0.01–10 μM; (C) IC_50_ curves and (D) IC_50_ values of the formulations GEM, VES–GEM, TPGS, TPGS/VES–GEM, and BxPC3 cell membrane-modified TPGS/VES–GEM micelles. (E) Cellular uptake of TPGS micelles showing cell nuclei dyed with DAPI and the micelles were dyed with Nile red (scale bar 50 μm). Controls were cells stained with Nile red and cells with culture medium. *p < 0.05, **p < 0.01, ***p < 0.001, and ****p < 0.0001.

The TPGS/VES–GEM (6/1) micelles were further tested regarding the ability to exert a cytotoxic effect in BxPC3 cell line and 3D model. For that, five different concentrations of VES–GEM were tested (0.01, 0.1, 1, 10, and 100 μM). Additionally, free VES–GEM and equivalent GEM concentrations were also compared with the formulation (Figure 7(A,B); Table S1).

For concentrations equal or higher to 1 μM, both free VES–GEM and TPGS/VES–GEM (6/1) micelles showed cytotoxic effect able to decrease cell viability below 50% in the 2D model. When concentrations of VES–GEM increased to 10 μM and 100 μM, the TPGS/VES–GEM (6/1) micelles showed the best cytotoxic activity of all groups and elicited significantly higher cytotoxic activity when compared to free VES–GEM (Figure 7(A), Table S2). This is probably due to the combination of anticancer activity displayed by TPGS which is seen extensively for the blank TPGS group (Khare et al. 2016). At both 10 μM and 100 μM, the cells treated with blank TPGS and TPGS/VES–GEM (6/1) micelles showed similar effects (*p* > .05) on cell viability, close to 0%. This finding is related to that at the observed concentrations of TPGS (1.17 mg/mL for 100 μM and 0.117 mg/mL for 10 μM), TPGS alone exhibited significant cytotoxic activity and was capable of eradicating PC cells in the 2D model without the need for combination with VES–GEM. This further corroborates the anticancer activity of TPGS (for concentrations above 0.1% w/v) and possibly VES (Khare et al. 2016). Some studies explored lower TPGS concentrations in cellular assays, often below 0.1% w/v, where its cytotoxic effects are limited and do not incorporate TPGS blank micelles as control, which limits study comparability. In our system, the ∼1.17 mg/mL TPGS concentration likely exceeds the functional threshold for bioactivity, explaining the blank micelle cytotoxicity observed at higher doses, and in agreement with other studies (Chary et al. 2024; Dian et al. 2024). It is also possible that some cancer cell lines such as BxPC3 may be particularly sensitive to oxidative or membrane-disrupting activity of TPGS, which could magnify the observed cytotoxic effect of TPGS compared to other reported cancer cell lines. Nonetheless IC_50_ values were significantly lower for TPGS/VES–GEM group (∼0.7 μM) when compared to TPGS (∼4.2 μM) (Figure 7(C,D)). When concentration range decreased, the combination effect between TPGS and VES–GEM (0.1 μM and 1 μM) became more evident. Also interestingly, at higher concentrations of VES–GEM the TPGS/VES–GEM (6/1) micelles demonstrated superior anticancer activity when compared to standard chemotherapy agent GEM, likely due to enhanced stability, improved cell internalization and combinatory effect with the bioactive building blocks. Another possible contribution to the significant cytotoxic activity of the TPGS/VES–GEM micelles in BxPC3 cells may be attributed to the enhanced stability observed to the capability to encapsulate hydrophobic VES–GEM and the strong ability to retain the prodrug evidenced by the limited VES–GEM release throughout the duration of the experiment. The strong hydrophobic nature of VES–GEM may contribute to its EE and enable interaction with VE segment of TPGS.

The GEM prodrug was less cytotoxic than GEM in the 2D model probably due to the chemical stability of the linker – an amide bond – formed between GEM and VES and the slow release of GEM from the prodrug system at the conditions observed in the experiment, as observed before in similar range of concentrations and GEM–lipid derivative strategies for PC therapy (Daman et al. 2014).

The ability of TPGS micelles to improve cytotoxic potential of anticancer drugs have been widely described on account of improved drug solubility and stability, maximized cellular uptake and superior therapeutic index when compared to the free drug (Wang et al. 2014; Xu et al. 2015; Khare et al. 2016; Yuan et al. 2018). The enhanced anticancer effect in both TPGS and TPGS/VES–GEM groups may be attributed to the MDR reversal activities attributed to TPGS and VE segment and through several pathways including P-gp (Liu, Wu, et al. 2016; Chen et al. 2023) and ATPase (Ma et al. 2014) inhibition. Thus, TPGS has been described to act as an effective cell uptake enhancer and enable improving the performance of poorly water-soluble anticancer drugs (Rathod et al. 2021; Mehata et al. 2023). Furthermore, it is expected that VE can be released from TPGS backbone through hydrolytic cleavage assisted by esterases present in the biological milieu, which can additionally contribute to overall anticancer effect (Rathod et al. 2021; Mehata et al. 2023). Interestingly, BxPC3 cell membrane-modified TPGS/VES–GEM micelles showed no statistical significant differences when compared to non-modified ones for 10 μM group, and may be due to majority of TPGS action and to the increased stability of the modified system. TPGS and VES are both largely explored materials with established biocompatibility and have been widely used in drug delivery systems with minimal toxicity to normal cells at the concentrations in the range of those used in this work (Yuan et al. 2018; Tang et al. 2023). Therefore, based on the selective cytotoxicity observed in some cancer cell lines versus normal cell lines reported in literature (Neophytou et al. 2014, 2019; Chen et al. 2020; Fan et al. 2021), including BxPC-3, we anticipate that the formulation will exhibit a favorable biocompatibility profile in normal tissues. Nonetheless, further *in vivo* studies are needed to confirm this selectivity and safety profile under physiological conditions.

The formulations were further explored in a 3D model with higher complexity and ability to display superior physiological relevance. A 3D *in vitro* model composed of cell-laden hydrogel tumoroids was developed according to previous literature. The results (Figure 7(B), Table S3) showed that, for the highest concentration point tested (100 µM), both the VES–GEM loaded and blank formulations exhibited a remarkable ability to decrease cell viability when compared with the free VES–GEM group demonstrating the notorious anticancer effect of TPGS polymer. This also suggested the ability of the TPGS micelles to penetrate in the tumoroid and pose as suitable PC-targeted systems aided by their enhanced stability and small size. The ability of TPGS micelles to penetrate in 3D models and exert significant anticancer effect has been shown before and may be related to their reduced size, PEGylated hydrophilic coat, drug encapsulation, and delivery properties in combination with intrinsic multifunctional anticancer effect of VE and TPGS (Wang et al. 2015; He et al. 2020; Xu et al. 2021). The ability to accumulate in 3D tumor models may be further enhanced by selection of targeting ligands or even by manipulation of physicochemical properties of TPGS-modified polymers. Unexpectedly, BxPC3 cell membrane-modified TPGS/VES–GEM micelles showed equal or even less cell cytotoxicity (Figure S6, Table S1), suggesting the membrane modification may impact both stability, affinity, and VES–GEM release, and also can reduce the interaction between micelle system and cells on account of electrostatic repulsion, as the BxPC3 nanovesicles and modified system showed strongly negative ZP. Furthermore, the ability to exert anticancer action is also limited by the ability of the system to diffuse and penetrate the tumoroids (Belli et al. 2017; Chen, Wang, et al. 2024; Cybulski et al. 2024), which may be reduced in the case of the cell membrane-modified micelles. It would be expected the modified system to increase the interaction with receptors on the BxPC3 cells mainly through homotypic and active targeting in the case of a partial or total coating; however, the final obtained structure is not conclusive.

The cellular uptake of TPGS micelles was mild (Figure 7(E)) as some fluorescence could be observed and suggested partial internalization of the drug-loaded micelles. Additionally, it is possible that TPGS itself may not efficiently encapsulate Nile red and the results may be underestimated. The concentration of Nile red employed in this study was kept as low as reasonably possible, and the formulations were filtered through a hydrophilic PTFE syringe filter (25 mm, 0.4 µm) to remove unincorporated Nile red following previously established protocols (Butt et al. 2015; Pereira-Silva, Diaz-Gomez, et al. 2024). Some TPGS/VES–GEM micelles can be internalized and display cytotoxic activity, and the non-internalized ones may release VES–GEM at extracellular environment and the conjugate may penetrate easier in the cell membrane due to its lipophilic character, and exert additional cytotoxic effect (Pereira-Silva, Miranda-Pastoriza, et al. 2024). Several studies have reported efficient cellular internalization of TPGS-based micelles for cancer therapy (Wang et al. 2014; Xu et al. 2015; Khare et al. 2016; Liu, Wu, et al. 2016; Yuan et al. 2018) and the process mediating TPGS-based systems internalization, although still underexplored, has been discussed to involve multivalent mechanisms such as endocytosis- and micropinocytosis-dependent internalization (Cheng et al. 2018; Liu et al. 2018), and energy-dependent, caveolae-mediated endocytosis (Gu et al. 2016). Additionally, the VE portion of the TPGS polymer may be exposed and interact with receptors expressed at the surface of cancer cells and promote receptor-mediated endocytosis (Takada and Suzuki 2010; Tan et al. 2017). We hypothesize that the main mechanism of internalization of the TPGS micelles, with and without the BxPC3 membrane coating, may be the transcellular pathway such as clathrin- or caveolae-mediated endocytosis, consistent with their nanoscale dimensions (30–150 nm). Nevertheless, the presence of the membrane coating could facilitate the membrane interaction, as incorporates surface proteins that may improve homotypic interactions with source BxPC3 cells, thus potentially increasing micelle binding and internalization, enhancing the uptake of the system and the cytotoxicity accordingly.

The biocompatibility and drug delivery efficiency of TPGS and VES have been extensively reported in previous studies, supporting their use as components of the systems to enhance drug solubility and tumor-targeting properties (Tan et al. 2017; Yang et al. 2018; Kumbhar et al. 2022; Mehata et al. 2023). Furthermore, the metabolic pathways of GEM have been extensively studied (Han et al. 2022). Here, we report significant findings to provide a strong foundation for further investigations, including future *in vivo* studies necessary to assess the pharmacokinetics and therapeutic effectiveness of the proposed drug delivery systems.

## 4. Conclusions

Altogether, TPGS micelles loaded with a biofunctional GEM derivative composed of VES–GEM were investigated as viable GEM delivery vehicles for enhanced PC therapy. Using the facile and *green* solvent evaporation procedure, TPGS/VES–GEM (6/1) micelles were successfully developed and characterized. The micelles were optimized regarding molar ratio to yield ultra small size (∼30 nm), slightly negative surface charge and showed high encapsulation effectiveness (>95%). Purification procedures provided insights regarding the composition and integrity of the micelles and attested the uneasiness of these systems to undergo complex purification protocols. The formulation showed improved VES–GEM solubility, stability and release profile and was able to retain most of VES–GEM after seven days (∼70%). *In vitro* results in PC cell line BxPC3 showed excellent cytotoxic activity for the concentrations tested of 10 μM, 100 μM for both VES–GEM-loaded and blank TPGS micelles, and demonstrated the anticancer activities of TPGS as multifunctional building block toward fabrication of all-functional systems for PC therapy, despite the only modest cell internalization observed. Further biomimetic functionalization of TPGS/VES–GEM (6/1) micelle cores with BxPC3 nanovesicles resulted in structures with size ∼150 nm, ZP ∼−30 mV and PDI ∼0.2 following an extrusion approach. The obtained system composed of BxPC3 cell membrane-enriched TPGS/VES–GEM (6/1) micelles could possibly expand the therapeutic avenues of PC but demand the combination of more advanced techniques to ensure that the whole surface repertoire of ligands present in the membrane fragments are correctly retained (in amount and orientation) when mixed with the micelles.

## Supplementary Material

Supplementary.docx

## Data Availability

The authors confirm that the data supporting the findings of this study are available within the article and its supplementary materials. The data that support the findings of this study are available from the corresponding author, [C. A-L.], upon reasonable request.
