# Supplementary material for: Biomimetic cancer cell membrane-enriched vitamin E-stapled gemcitabine-loaded TPGS micelles for pancreatic cancer therapy
Source: Drug Deliv. 2025 Jul 9;32(1):2527759. doi: 10.1080/10717544.2025.2527759 (PMC12243015; doi:10.1080/10717544.2025.2527759)
Supplement: Supplementary.docx [file IDRD_A_2527759_SM3084.docx]

**SUPPLEMENTARY DATA**

***Biomimetic cancer cell membrane-enriched vitamin E-stapled gemcitabine-loaded TPGS micelles for pancreatic cancer therapy***

Miguel Pereira-Silva ^a,b,c^, Luis Diaz-Gomez ^c^, Bárbara Blanco-Fernandez ^c^, Ana Cláudia Paiva-Santos ^a,b^, Francisco Veiga ^a,b^, Angel Concheiro ^c^, Carmen Alvarez-Lorenzo ^c*^

^a^ Department of Pharmaceutical Technology, Faculty of Pharmacy, University of Coimbra, 3000-548 Coimbra, Portugal

^b^ REQUIMTE/LAQV, Group of Pharmaceutical Technology, Faculty of Pharmacy, University of Coimbra, 3000-548 Coimbra, Portugal

^c^ Departamento de Farmacología, Farmacia y Tecnología Farmacéutica, I+D Farma, Facultad de Farmacia, Instituto de Materiales (iMATUS) and Health Research Institute of Santiago de Compostela (IDIS), Universidade de Santiago de Compostela, 15782 Santiago de Compostela, Spain

***CORRESPONDING AUTHOR:**

* Carmen Alvarez-Lorenzo

E-mail: [carmen.alvarez.lorenzo@usc.es](mailto:carmen.alvarez.lorenzo@usc.es)


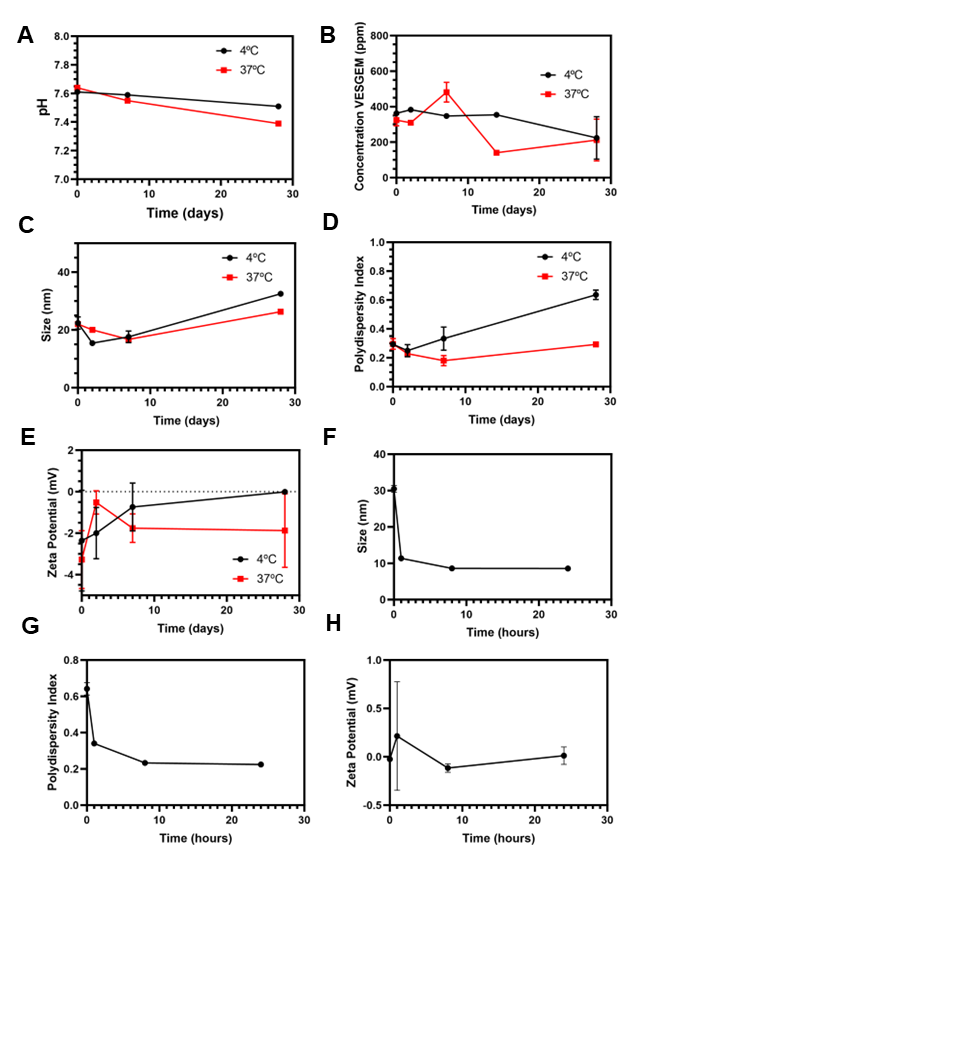


**Figure S1.** Stability evaluation of TPGS/VES-GEM (6/1) micelles prepared in PBS:water (50:50 *v/v*). pH (A), VES-GEM content (B), size (C), PDI (D), and (E) ZP of TPGS/VES-GEM (6/1) micelles at 4 ºC and 37 ºC, throughout 4 weeks. Size (F), PDI (G) and ZP (H) of TPGS micelles (prepared in PBS:water 50:50 *v/v*), diluted 1:9 in aqueous BSA solution (8 mg/mL) after stirring at RT, and aliquots collected at time = 0, 1, 8 and 24 h.

**
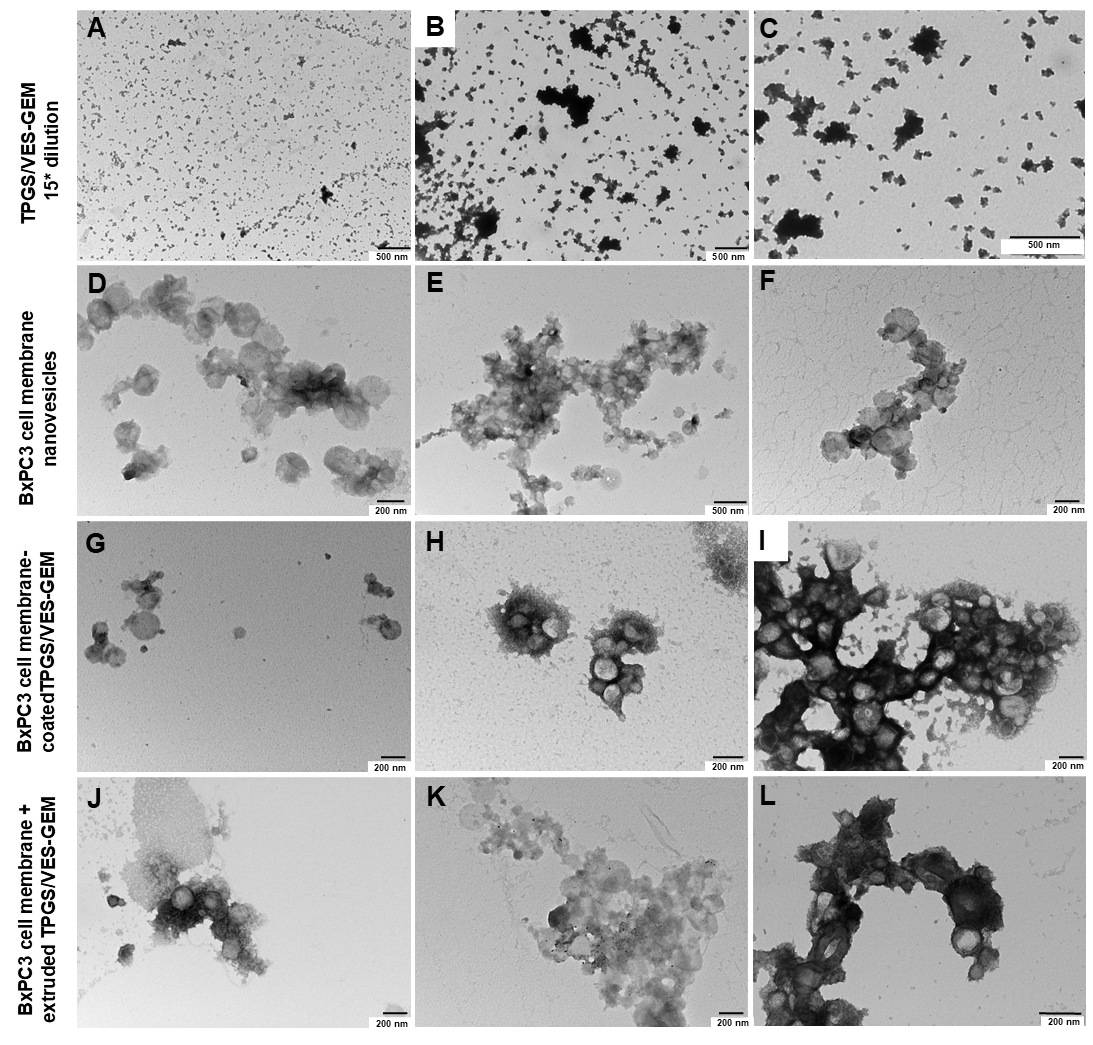
**

**Figure S2.** TEM pictures of the TPGS/VES-GEM micelles (polymer concentration of 0.22 mg/mL) (A-C), BxPC3 nanovesicles (D-F), BxPC3 nanovesicle-coated TPGS/VES-GEM micelles (polymer-to-protein ratio of 1:1) (G-I) and mixture of BxPC3 nanovesicles and extruded TPGS/VES-GEM micelles (1:4 dilution in water) following same polymer-to-protein membrane ratio. (J-L). Three photos were taken independently from the same prepared sample for each formulation. The samples were not filtered. Samples were stained with uranyl acetate 2%.


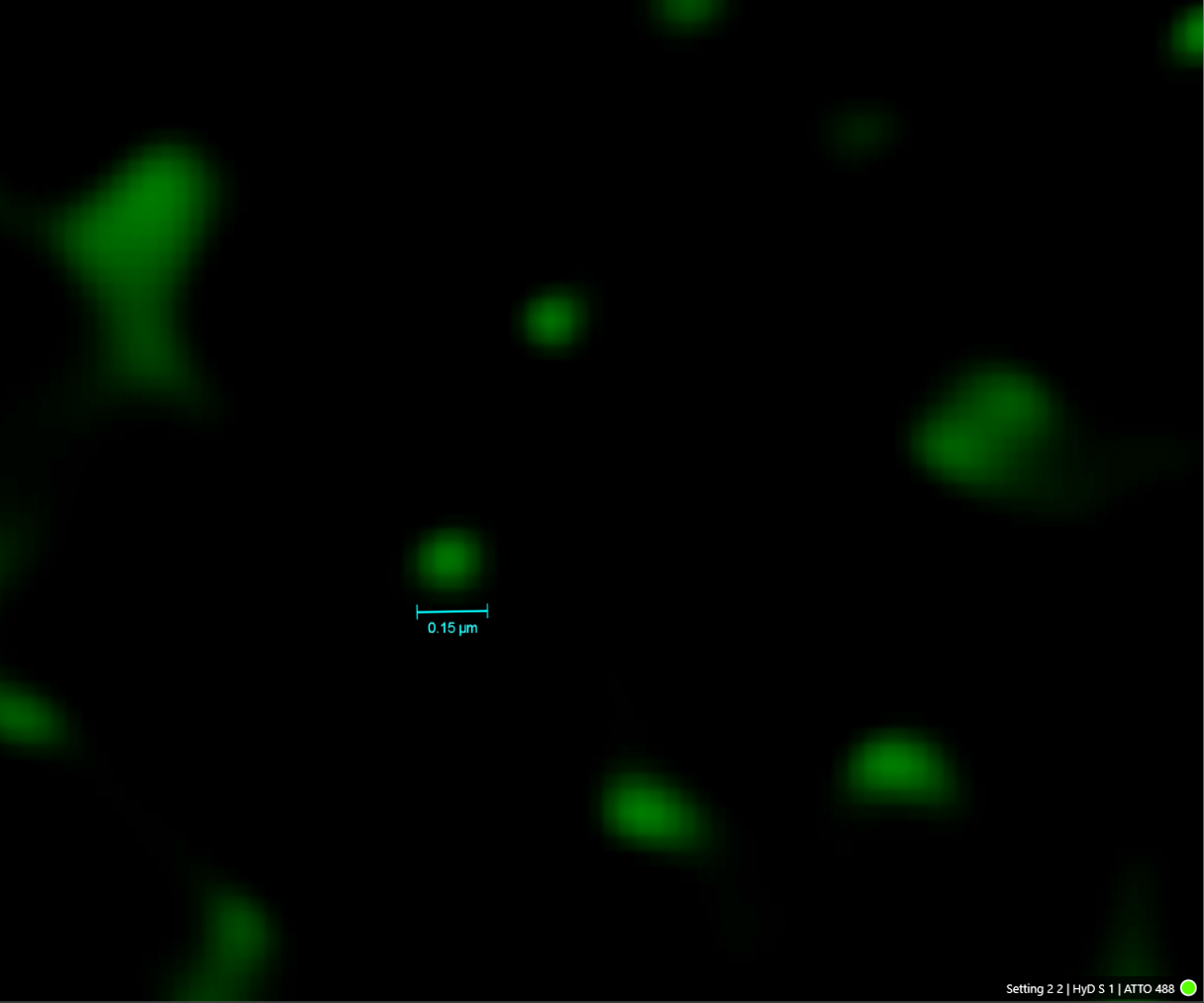


**Figure S3.** CLSM picture of BxPC3 cell membrane-coated TPGS micelles. Blank TPGS micelles were dyed with Nile red and DOPE-Atto 488was used to label BxPC3 nanovesicles through lipid bilayer intercalation. The formulation was prepared in PBS medium. CLSM pictures were taken using a Leica confocal TCS-SP5 microscope.

**
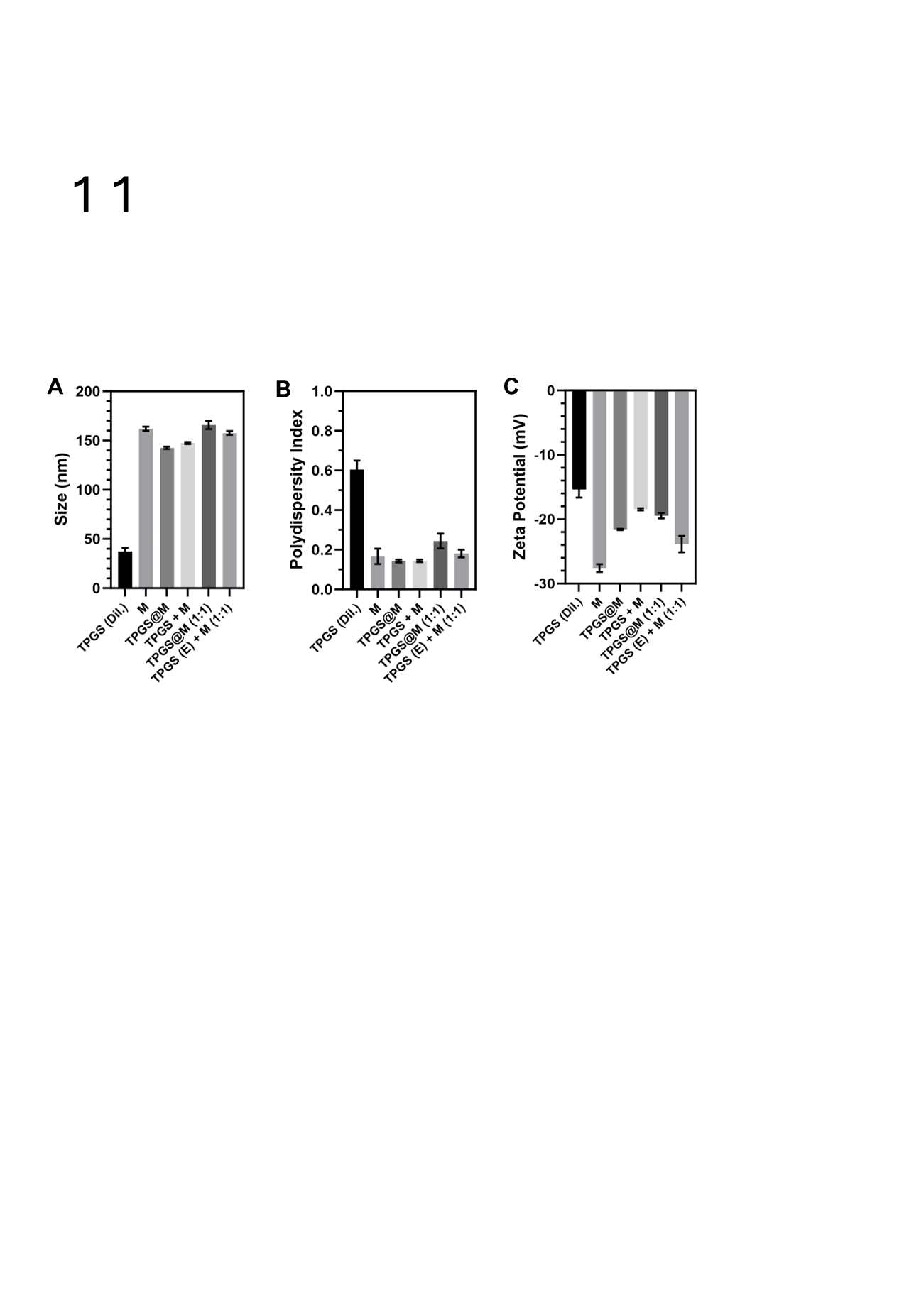
**

**Figure S4.** Size (A), PDI (B) and ZP (C) of TPGS/VES-GEM micelles 1:14 dilution (polymer concentration of 0.22 mg/mL, TPGS (Dil.)), BxPC3 nanovesicles (M), BxPC3 membrane-coated TPGS/VES-GEM micelles (polymer-to-protein ratio of 2:1, TPGS@M), mixture of BxPC3 nanovesicles and TPGS/VES-GEM micelles (polymer concentration of 0.63 mg/mL, 1:4 dilution in water, polymer-to-protein ratio of 2:1), TPGS+M, BxPC3 membrane-coated TPGS/VES-GEM micelles (polymer-to-protein ratio of 1:1, TPGS@M 1:1) and mixture of BxPC3 nanovesicles and extruded TPGS/VES-GEM micelles (polymer concentration of 0.63 mg/mL, 1:4 dilution in water, polymer-to-protein ratio of 1:1).

**
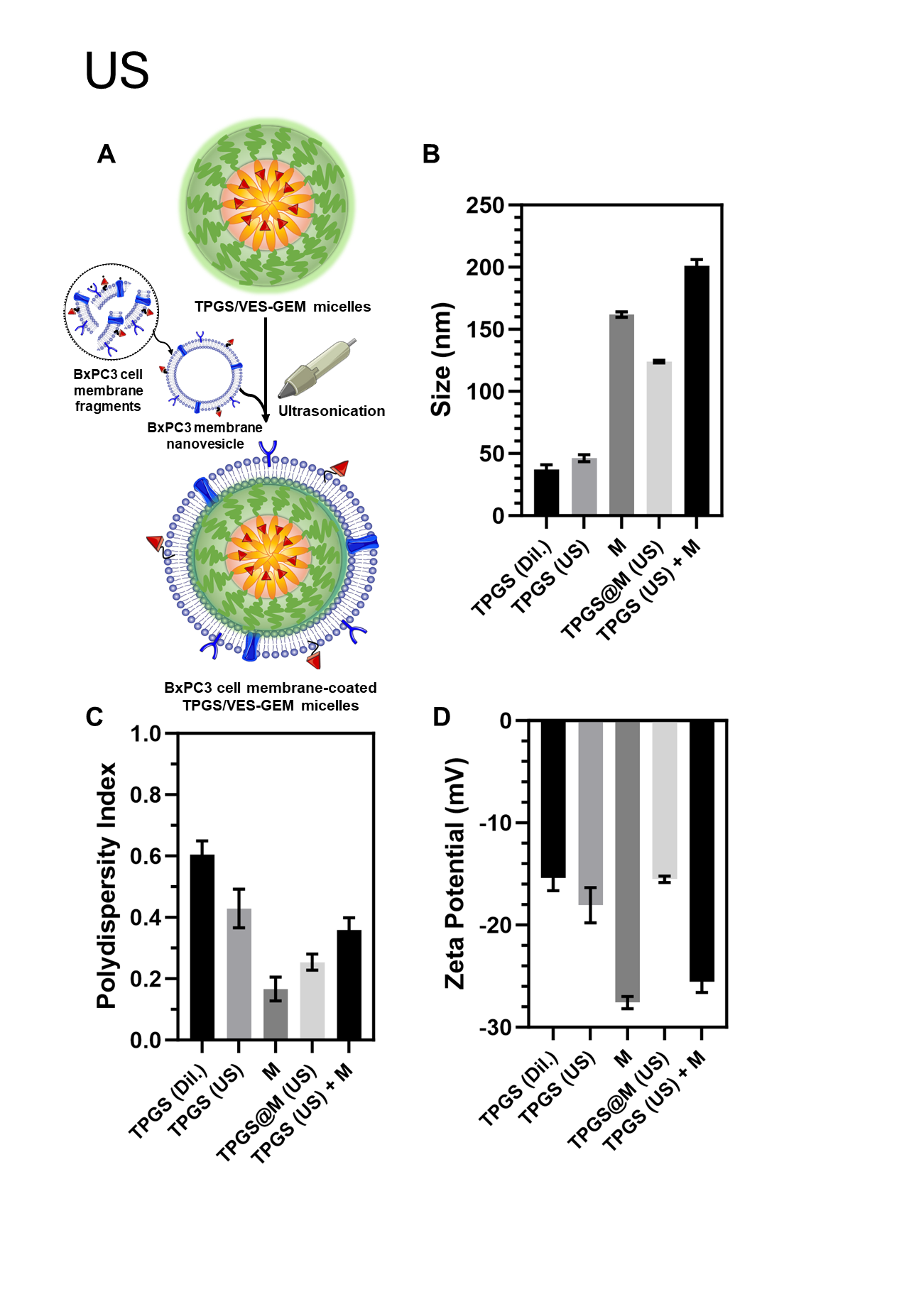
**

**Figure S5.** Schematic illustration of coating process of TPGS/VES-GEM micelle cores with BxPC3 cell membrane nanovesicles through ultrasonication (A). The functionalized micelles were prepared by first producing BxPC3 cell membrane nanovesicles, which were then added to TPGS/VES-GEM micelle formulation and co-ultrasonicated at polymer-to-protein ratio of 2:1 *w/w*. Size (B), PDI (C) and ZP (D) of TPGS/VES-GEM micelles 1:14 dilution (TPGS (Dil.)), TPGS/VES-GEM micelles ultrasonicated and diluted (1:14 final dilution), BxPC3 nanovesicles (M), BxPC3 membrane-coated TPGS/VES-GEM micelles (polymer-to-protein ratio of 2:1, TPGS@M (US)), and mixture of BxPC3 nanovesicles with ultrasonicated TPGS/VES-GEM micelles (polymer concentration of 0.63 mg/mL, 1:4 dilution in water, polymer-to-protein ratio of 2:1), TPGS(US)+M.


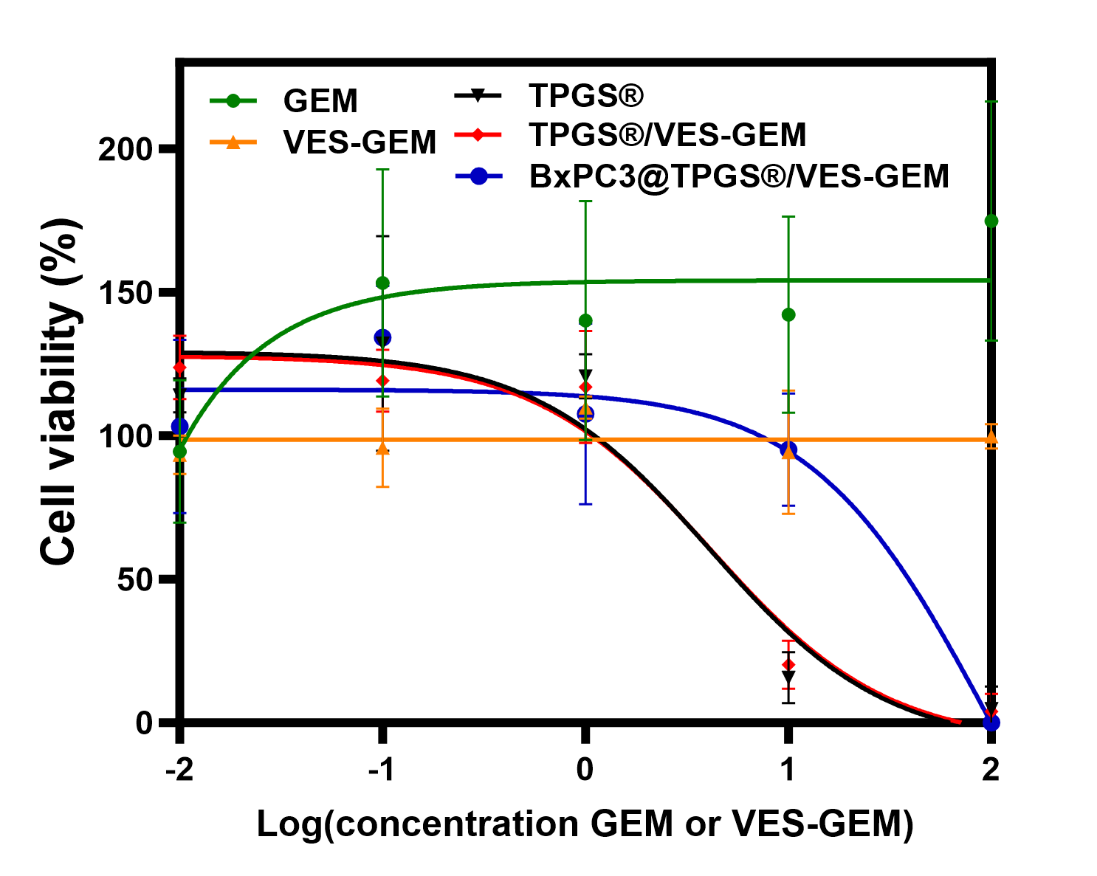


**Figure S6.** IC_50_ curves of the formulations GEM, VES-GEM, TPGS, TPGS/VES-GEM and BxPC3 cell membrane-modified TPGS/VES-GEM micelles.

**Table S1.** IC50 3D of the formulations GEM, VES-GEM, TPGS, TPGS/VES-GEM and BxPC3 cell membrane-modified TPGS/VES-GEM micelles.

| **Formulation** | **IC50 (µM)** |
| --- | --- |
| **GEM** | - |
| **VES-GEM** | - |
| **TPGS** | 4.101 |
| **TPGS/VES-GEM** | 4.150 |
| **BxPC3@TPGS/VES-GEM** | 95.16 |

**Table S2.** Statistical significance table for cell viability in *in vitro* BxPC3 cell line model, using two-way ANOVA, Tukey’s multiple comparisons test. Data obtained from GraphPad Prism 10.1.2.

| **Formulation / log(µM)** | **Mean Diff.** | **95.00% CI of diff.** | **Below threshold?** | **Summary** | **Adjusted P Value** |
| --- | --- | --- | --- | --- | --- |
| **0.01** |  |  |  |  |  |
| GEM vs. VES-GEM | -16.82 | -28.63 to -5.023 | Yes | ** | 0.0013 |
| GEM vs. TPGS | -14.47 | -26.27 to -2.671 | Yes | ** | 0.0082 |
| GEM vs. TPGS/VES-GEM | -36.85 | -48.65 to -25.05 | Yes | **** | <0.0001 |
| GEM vs. BxPC3@TPGS/VES-GEM | -43.48 | -55.28 to -31.68 | Yes | **** | <0.0001 |
| VES-GEM vs. TPGS | 2.352 | -9.449 to 14.15 | No | ns | 0.9812 |
| VES-GEM vs. TPGS/VES-GEM | -20.02 | -31.83 to -8.224 | Yes | **** | <0.0001 |
| VES-GEM vs. BxPC3@TPGS/VES-GEM | -26.66 | -38.46 to -14.86 | Yes | **** | <0.0001 |
| TPGS vs. TPGS/VES-GEM | -22.38 | -34.18 to -10.58 | Yes | **** | <0.0001 |
| TPGS vs. BxPC3@TPGS/VES-GEM | -29.01 | -40.81 to -17.21 | Yes | **** | <0.0001 |
| TPGS/VES-GEM vs. BxPC3@TPGS/VES-GEM | -6.634 | -18.44 to 5.167 | No | ns | 0.5253 |
| **0.1** |  |  |  |  |  |
| GEM vs. VES-GEM | -29.71 | -41.51 to -17.90 | Yes | **** | <0.0001 |
| GEM vs. TPGS | -69.41 | -81.21 to -57.61 | Yes | **** | <0.0001 |
| GEM vs. TPGS/VES-GEM | -50.98 | -62.78 to -39.18 | Yes | **** | <0.0001 |
| GEM vs. BxPC3@TPGS/VES-GEM | -82.18 | -93.98 to -70.38 | Yes | **** | <0.0001 |
| VES-GEM vs. TPGS | -39.71 | -51.51 to -27.90 | Yes | **** | <0.0001 |
| VES-GEM vs. TPGS/VES-GEM | -21.27 | -33.07 to -9.472 | Yes | **** | <0.0001 |
| VES-GEM vs. BxPC3@TPGS/VES-GEM | -52.48 | -64.28 to -40.68 | Yes | **** | <0.0001 |
| TPGS vs. TPGS/VES-GEM | 18.43 | 6.630 to 30.23 | Yes | *** | 0.0003 |
| TPGS vs. BxPC3@TPGS/VES-GEM | -12.77 | -24.57 to -0.9707 | Yes | * | 0.0270 |
| TPGS/VES-GEM vs. BxPC3@TPGS/VES-GEM | -31.20 | -43.00 to -19.40 | Yes | **** | <0.0001 |
| **1** |  |  |  |  |  |
| GEM vs. VES-GEM | -27.07 | -38.87 to -15.27 | Yes | **** | <0.0001 |
| GEM vs. TPGS | -79.66 | -91.47 to -67.86 | Yes | **** | <0.0001 |
| GEM vs. TPGS/VES-GEM | -22.77 | -34.57 to -10.96 | Yes | **** | <0.0001 |
| GEM vs. BxPC3@TPGS/VES-GEM | -43.99 | -55.79 to -32.19 | Yes | **** | <0.0001 |
| VES-GEM vs. TPGS | -52.60 | -64.40 to -40.80 | Yes | **** | <0.0001 |
| VES-GEM vs. TPGS/VES-GEM | 4.301 | -7.500 to 16.10 | No | ns | 0.8490 |
| VES-GEM vs. BxPC3@TPGS/VES-GEM | -16.93 | -28.73 to -5.126 | Yes | ** | 0.0012 |
| TPGS vs. TPGS/VES-GEM | 56.90 | 45.10 to 68.70 | Yes | **** | <0.0001 |
| TPGS vs. BxPC3@TPGS/VES-GEM | 35.67 | 23.87 to 47.47 | Yes | **** | <0.0001 |
| TPGS/VES-GEM vs. BxPC3@TPGS/VES-GEM | -21.23 | -33.03 to -9.427 | Yes | **** | <0.0001 |
| **10** |  |  |  |  |  |
| GEM vs. VES-GEM | -14.67 | -26.47 to -2.864 | Yes | ** | 0.0071 |
| GEM vs. TPGS | 22.40 | 10.60 to 34.20 | Yes | **** | <0.0001 |
| GEM vs. TPGS/VES-GEM | 21.35 | 9.545 to 33.15 | Yes | **** | <0.0001 |
| GEM vs. BxPC3@TPGS/VES-GEM | 15.98 | 4.183 to 27.79 | Yes | ** | 0.0026 |
| VES-GEM vs. TPGS | 37.06 | 25.26 to 48.87 | Yes | **** | <0.0001 |
| VES-GEM vs. TPGS/VES-GEM | 36.01 | 24.21 to 47.81 | Yes | **** | <0.0001 |
| VES-GEM vs. BxPC3@TPGS/VES-GEM | 30.65 | 18.85 to 42.45 | Yes | **** | <0.0001 |
| TPGS vs. TPGS/VES-GEM | -1.053 | -12.85 to 10.75 | No | ns | 0.9992 |
| TPGS vs. BxPC3@TPGS/VES-GEM | -6.416 | -18.22 to 5.386 | No | ns | 0.5583 |
| TPGS/VES-GEM vs. BxPC3@TPGS/VES-GEM | -5.363 | -17.16 to 6.439 | No | ns | 0.7146 |
| **100** |  |  |  |  |  |
| GEM vs. VES-GEM | -21.75 | -33.55 to -9.952 | Yes | **** | <0.0001 |
| GEM vs. TPGS | 16.35 | 4.549 to 28.15 | Yes | ** | 0.0019 |
| GEM vs. TPGS/VES-GEM | 16.46 | 4.655 to 28.26 | Yes | ** | 0.0018 |
| VES-GEM vs. TPGS | 38.10 | 26.30 to 49.90 | Yes | **** | <0.0001 |
| VES-GEM vs. TPGS/VES-GEM | 38.21 | 26.41 to 50.01 | Yes | **** | <0.0001 |
| TPGS vs. TPGS/VES-GEM | 0.1064 | -11.69 to 11.91 | No | ns | >0.9999 |

**Table S3**. Statistical significance table for cell viability in *in vitro* 3D model. using two-way ANOVA. Tukey’s multiple comparisons test. Data obtained from GraphPad Prism 10.1.2.

| **Formulation / log(µM)** | **Mean Diff.** | **95.00% CI of diff.** | **Below threshold?** | **Summary** | **Adjusted P Value** |
| --- | --- | --- | --- | --- | --- |
| **0.01** |  |  |  |  |  |
| GEM vs. VES-GEM | 1.221 | -42.52 to 44.96 | No | ns | >0.9999 |
| GEM vs. TPGS | -19.43 | -63.17 to 24.31 | No | ns | 0.7270 |
| GEM vs. TPGS/VES-GEM | -29.31 | -73.05 to 14.44 | No | ns | 0.3406 |
| GEM vs. BxPC3@TPGS/VES-GEM | -8.697 | -52.44 to 35.04 | No | ns | 0.9809 |
| VES-GEM vs. TPGS | -20.65 | -64.39 to 23.09 | No | ns | 0.6798 |
| VES-GEM vs. TPGS/VES-GEM | -30.53 | -74.27 to 13.21 | No | ns | 0.3000 |
| VES-GEM vs. BxPC3@TPGS/VES-GEM | -9.918 | -53.66 to 33.82 | No | ns | 0.9690 |
| TPGS vs. TPGS/VES-GEM | -9.879 | -53.62 to 33.86 | No | ns | 0.9695 |
| TPGS vs. BxPC3@TPGS/VES-GEM | 10.73 | -33.01 to 54.47 | No | ns | 0.9589 |
| TPGS/VES-GEM vs. BxPC3@TPGS/VES-GEM | 20.61 | -23.13 to 64.35 | No | ns | 0.6813 |
| **0.1** |  |  |  |  |  |
| GEM vs. VES-GEM | 57.38 | 13.64 to 101.1 | Yes | ** | 0.0041 |
| GEM vs. TPGS | 21.08 | -26.17 to 68.32 | No | ns | 0.7237 |
| GEM vs. TPGS/VES-GEM | 34.02 | -9.723 to 77.76 | No | ns | 0.2008 |
| GEM vs. BxPC3@TPGS/VES-GEM | 19.03 | -24.71 to 62.78 | No | ns | 0.7417 |
| VES-GEM vs. TPGS | -36.31 | -83.55 to 10.94 | No | ns | 0.2110 |
| VES-GEM vs. TPGS/VES-GEM | -23.37 | -67.11 to 20.38 | No | ns | 0.5698 |
| VES-GEM vs. BxPC3@TPGS/VES-GEM | -38.35 | -82.09 to 5.392 | No | ns | 0.1133 |
| TPGS vs. TPGS/VES-GEM | 12.94 | -34.31 to 60.19 | No | ns | 0.9396 |
| TPGS vs. BxPC3@TPGS/VES-GEM | -2.043 | -49.29 to 45.20 | No | ns | >0.9999 |
| TPGS/VES-GEM vs. BxPC3@TPGS/VES-GEM | -14.98 | -58.72 to 28.76 | No | ns | 0.8729 |
| **1** |  |  |  |  |  |
| GEM vs. VES-GEM | 30.32 | -13.43 to 74.06 | No | ns | 0.3068 |
| GEM vs. TPGS | 19.43 | -24.31 to 63.17 | No | ns | 0.7269 |
| GEM vs. TPGS/VES-GEM | 23.20 | -20.54 to 66.94 | No | ns | 0.5765 |
| GEM vs. BxPC3@TPGS/VES-GEM | 32.61 | -11.13 to 76.35 | No | ns | 0.2378 |
| VES-GEM vs. TPGS | -10.89 | -54.63 to 32.86 | No | ns | 0.9568 |
| VES-GEM vs. TPGS/VES-GEM | -7.113 | -50.85 to 36.63 | No | ns | 0.9910 |
| VES-GEM vs. BxPC3@TPGS/VES-GEM | 2.292 | -41.45 to 46.03 | No | ns | 0.9999 |
| TPGS vs. TPGS/VES-GEM | 3.773 | -39.97 to 47.51 | No | ns | 0.9992 |
| TPGS vs. BxPC3@TPGS/VES-GEM | 13.18 | -30.56 to 56.92 | No | ns | 0.9164 |
| TPGS/VES-GEM vs. BxPC3@TPGS/VES-GEM | 9.405 | -34.34 to 53.15 | No | ns | 0.9745 |
| **10** |  |  |  |  |  |
| GEM vs. VES-GEM | 47.91 | 4.169 to 91.65 | Yes | * | 0.0247 |
| GEM vs. TPGS | 126.4 | 82.68 to 170.2 | Yes | **** | <0.0001 |
| GEM vs. TPGS/VES-GEM | 121.9 | 78.19 to 165.7 | Yes | **** | <0.0001 |
| GEM vs. BxPC3@TPGS/VES-GEM | 47.10 | 3.362 to 90.85 | Yes | * | 0.0284 |
| VES-GEM vs. TPGS | 78.52 | 34.77 to 122.3 | Yes | **** | <0.0001 |
| VES-GEM vs. TPGS/VES-GEM | 74.02 | 30.28 to 117.8 | Yes | **** | <0.0001 |
| VES-GEM vs. BxPC3@TPGS/VES-GEM | -0.8063 | -44.55 to 42.94 | No | ns | >0.9999 |
| TPGS vs. TPGS/VES-GEM | -4.495 | -48.24 to 39.25 | No | ns | 0.9985 |
| TPGS vs. BxPC3@TPGS/VES-GEM | -79.32 | -123.1 to -35.58 | Yes | **** | <0.0001 |
| TPGS/VES-GEM vs. BxPC3@TPGS/VES-GEM | -74.83 | -118.6 to -31.08 | Yes | **** | <0.0001 |
| **100** |  |  |  |  |  |
| GEM vs. VES-GEM | 75.03 | 31.29 to 118.8 | Yes | **** | <0.0001 |
| GEM vs. TPGS | 170.0 | 126.2 to 213.7 | Yes | **** | <0.0001 |
| GEM vs. TPGS/VES-GEM | 170.9 | 127.2 to 214.7 | Yes | **** | <0.0001 |
| VES-GEM vs. TPGS | 94.93 | 51.19 to 138.7 | Yes | **** | <0.0001 |
| VES-GEM vs. TPGS/VES-GEM | 95.89 | 52.15 to 139.6 | Yes | **** | <0.0001 |
| TPGS vs. TPGS/VES-GEM | 0.9620 | -42.78 to 44.70 | No | ns | >0.9999 |
